# Decrypting a cryptic allosteric pocket in *H. pylori* glutamate racemase

**DOI:** 10.1038/s42004-021-00605-z

**Published:** 2021-12-10

**Authors:** Pratik Rajesh Chheda, Grant T. Cooling, Sondra F. Dean, Jonah Propp, Kathryn F. Hobbs, M. Ashley Spies

**Affiliations:** 1 Division of Medicinal and Natural Products Chemistry, Department of Pharmaceutical Sciences and Experimental Therapeutics, The University of Iowa, Iowa City, IA 52242 USA; 2 Department of Biochemistry, Carver College of Medicine, The University of Iowa, Iowa City, IA 52242 USA

**Keywords:** Structure-based drug design, Computational chemistry

## Abstract

One of our greatest challenges in drug design is targeting cryptic allosteric pockets in enzyme targets. Drug leads that do bind to these cryptic pockets are often discovered during HTS campaigns, and the mechanisms of action are rarely understood. Nevertheless, it is often the case that the allosteric pocket provides the best option for drug development against a given target. In the current studies we present a successful way forward in rationally exploiting the cryptic allosteric pocket of *H. pylori* glutamate racemase, an essential enzyme in this pathogen’s life cycle. A wide range of computational and experimental methods are employed in a workflow leading to the discovery of a series of natural product allosteric inhibitors which occupy the allosteric pocket of this essential racemase. The confluence of these studies reveals a fascinating source of the allosteric inhibition, which centers on the abolition of essential monomer-monomer coupled motion networks.

## Abstract (web summary)

Protein-ligand binding can induce the formation of cryptic allosteric pockets not found in the unbound protein, but predicting this computationally can be challenging. Here a combined computational and experimental workflow identifies ligands for *H. pylori*glutamate racemase, finding that coupled dynamics of the enzyme dimer are dampened by ligand binding.

## Introduction

*Helicobacter pylori* (*H. pylori*) is a spiral-shaped Gram-negative pathogenic bacterium estimated to infect more than half of the world’s population^1,2^*. H. pylori* infections may persist for a lifetime if treated inappropriately and have been associated with numerous gastrointestinal diseases like chronic gastritis, gastric ulcers, and several forms of gastric cancer^3,4^. Current treatment options involve the use of relatively broad antibiotics, which do not specifically target *H. pylori*^5,6^. The current work focuses on structure-based design targeting an essential enzyme in the *H. pylori* life cycle, glutamate racemase (GR), which catalyzes the stereo-inversion of glutamate. D-glutamate (D-Glu) is an essential component of the peptidoglycan layer of bacterial cell walls, which protects the microbe from osmotic rupture and proteolytic damage^7^. Importantly, although glutamate racemases are ubiquitous in the bacterial world, the *H. pylori* GR possesses a unique cryptic allosteric pocket, which has been the subject of robust drug discovery efforts, from both the pharmaceutical industry^8–12^ and academia^13–18^.

**Fig. 1:**
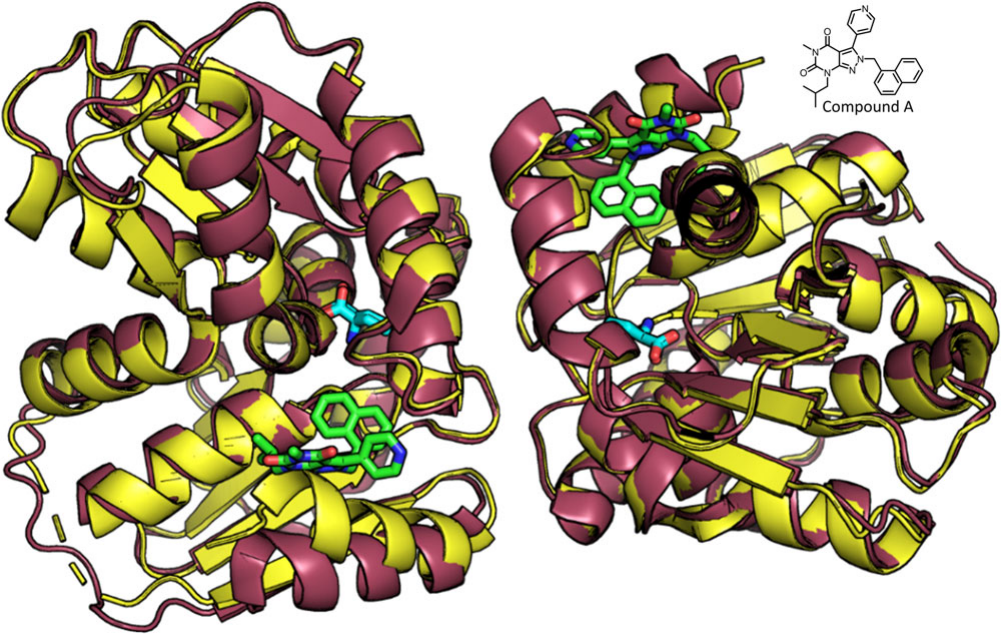
*H. pylori* GR dimer structure bound to D-Glu (substrate) and Compound A (allosteric inhibitor). *H. pylori* GR exists as an obligate dimer, with the active sites of each monomer facing one another (D-Glu is shown in the center of each monomer in blue and red). Structure PDB 2JFZ (brown ribbons) bound to Compound A (shown in green) bound to the allosteric pocket is superposed onto the inhibitor free form of the enzyme PDB 2JFX (yellow ribbons). Mechanistically, these structures alone are not sufficient to understand allosteric inhibition in this system, which is largely driveni by dynamics, as discussed in the text.

In one of the most successful studies to date, researchers at AstraZeneca performed a large high-throughput screen (of ~400 K compounds) and identified a single viable lead compound, a pyrazolopyrimidinedione (designated ‘Compound A’ – Fig. 1) as an uncompetitive allosteric inhibitor of *H. pylori* GR^8^. Lundqvist et al., reported that Compound A had an IC_50_ value of 5 μM, and X-ray crystallography studies showed it to be binding in a cryptic allosteric pocket remote from the active site (Fig. 1). Despite extensive optimization cycles, no clinical candidate emerged, due to poor solubility and high protein binding for this class of compounds^9,10,12^. Thus, despite being a validated antibiotic drug target, there is no clinically available inhibitor of *H. pylori* GR. A compounding factor is that a mechanism of action, by which occupancy of the cryptic allosteric pocket remotely leads to inhibition has never been elucidated. Indeed, it is difficult to imagine gaining medicinal chemistry traction on this target in the absence of such knowledge. This is a trend that has plagued allosteric drug discovery in general^19,20^. In Lundquist et al., the authors hypothesized that uncompetitive inhibition occurs due to Compound A binding via limiting a hinge movement in each monomer, thus restricting substrate-product release^8^. Our group recently disproved the proposed uncompetitive kinetic mechanism of *H. pylori* GR, and proposed an alternative mechanism of action, based on computational and experimental data^15^. MD and QM/MM studies in Witkin et al., found that *H. pylori* GR goes through a large conformational change that facilitates the acidification of the substrate Cα-proton via a protonation of the α-carboxylate oxygen from the catalytic Cys185; the entire process is a key part of a pre-activation step that occurs before the racemization catalytic cycle (Supplementary Fig. 1)^15^. However, essentially nothing is known about how occupancy of the allosteric pocket by this inhibitor promotes dampening of the chemical activation process that makes the extraordinarily difficult chemistry of racemization possible.

An important scientific and pharmaceutical question is whether or not structure based discovery approaches can be successfully applied to a cryptic allosteric pocket, as in the case of *H. pylori* GR, especially given our limited understanding of the manifold forms that such a pocket may take; it is particularly problematic due to the global flexibility of GR and a lack of experimental structural data about the apo form of the enzyme^7^. Although allosteric drugs hold enormous promise, the truth is that only 19 of more than 3700 FDA approved drugs are allosteric, and only a single drug was derived using approaches centering on in silico/structural methods^20^. These are extremely challenging problems, which necessitate the use of methods that stress test our computational and structural approaches. In this study we report an MD/Docking workflow which is stress tested via the Receiver Operator Characteristic (ROC) statistical method, and results in the successful discovery of a new allosteric inhibitor of *H. pylori* GR, but with a completely different chemical space than Compound A, proving that the workflow presented herein is able to address the challenges presented by cryptic allosteric pockets. Importantly, the MD-based discovery process itself reveals a distinct allosteric communication in the native system (no inhibitor) which occurs between monomers of the dimeric enzyme via a dynamic C-terminal α-helix, which is dramatically dampened by complexation with the allosteric inhibitor. The dynamical data is strongly supported by the pattern in the crystallographic B-factors in the inhibited and uninhibited complexes of *H. pylori* GR, which has not been previously recognized. These findings constitute a definitive and novel type of Allosteric Structure Activity Relationship (ASAR).

## Results

### Overall summary of structure-based discovery workflow for cryptic allosteric pocket

**Fig. 2:**
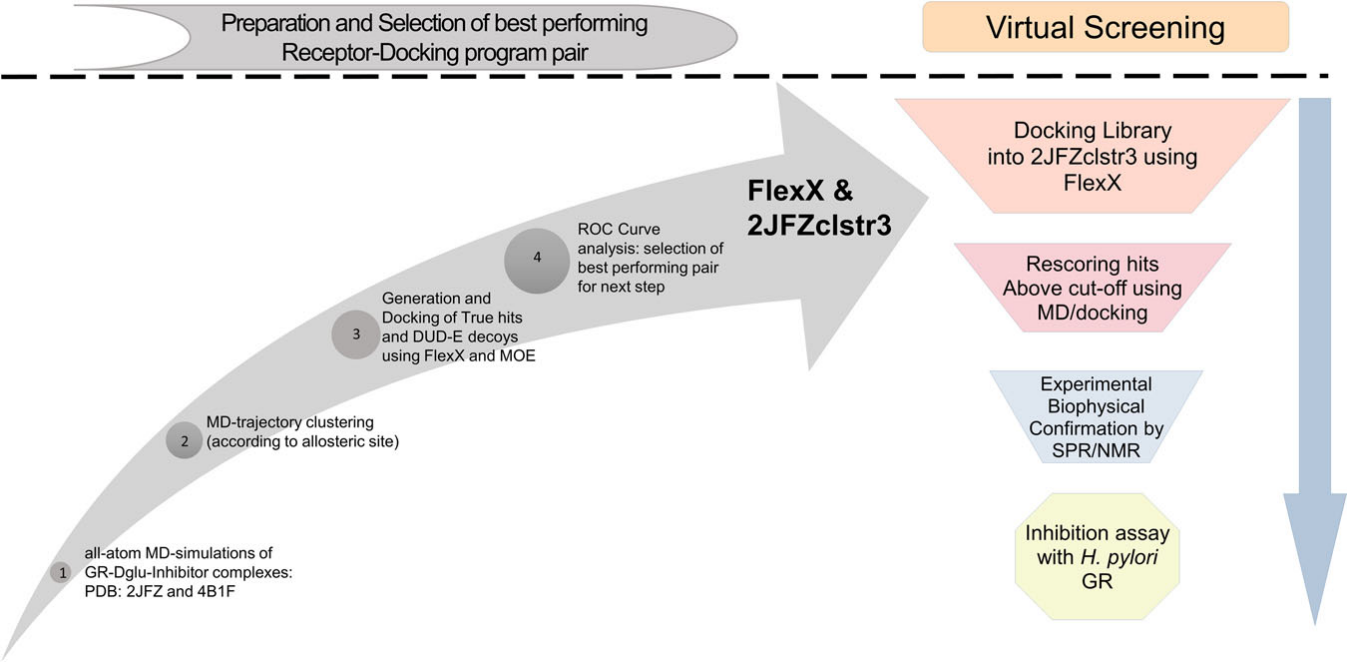
Biphasic workflow for natural product inhibitor discovery targeting the allosteric cryptic pocket of *H. pylori GR*. The graph is divided into two phases, depicted on the left and the right, respectively. On the left half of the chart, we describe the selection of the best receptor and docking protocol combination; snapshots from MD simulations of ~800 ns were clustered and centroids were examined for their ROC performance (ability to discriminate between decoys and known hits). The best performing pair was “*2JFZclstr3*” as the receptor (derived from the PDB structure **2JFZ**, as described in the Methods section) and docking was performed with FlexX (BiosolveIT), as described in the Methods section. The flowchart on the right-hand side is the actual virtual screening protocol that employs the validated *2JFZclstr3*-FlexX (receptor and docking protocol) pair, including the experimental biophysical hit validation using SPR. The screening library employed was AnalytiCon’s MEGx natural products library.

In the current study we report a virtual screening regime developed specifically for highly flexible enzyme targets like *H. pylori* GR and which contain a cryptic allosteric pocket (Fig. 2). Here we briefly describe the broad outlines and philosophy of this approach, while a more detailed description can be found in the Methods section. The central feature of this approach involves selection of the “best-performing” combination of receptor (from MD trajectories) and docking protocol/program for virtual screening. The workflow employs a hybrid Molecular Dynamics-Docking approach involving all atom classical MD simulations followed by global structural clustering of MD snapshots to generate different receptors forms. Known actives and decoys were docked into each form of the receptors using different docking protocols. The results were then analyzed using the statistical analysis technique of Receiver Operating Characteristic (ROC) curves^21–24^, to select the best performing MD-receptor/docking protocol pair (defined in the ROC approach as its ability to separate true positives from false positives given a user-defined selection threshold value (i.e., docking score), which will be elaborated on below) (Fig. 2). Due to the very low hit rates obtained in HTS screening against *H. pylori* GR using drug-like synthetic compounds observed by Lundqvist et al.^8^, and the solubility challenges with Compound A,^9,10,12^ along with the aim to challenge our workflow with unconventional chemical matter, we chose to focus on in silico screening of natural product libraries. In silico screening of natural product libraries, which are commercially available in a purified form, have recently been used to great effect against difficult drug targets^24^. Importantly, natural products provide an unrivaled success rate in drug lead discovery^25–27^, but have been under-exploited in in silico approaches, often due to the difficulty and expense in obtaining purified compounds. Thus, the best performing MD-receptor/docking protocol pair was employed to screen the AnalytiCon’s MEGx natural products library, which resulted in 177 hits that scored better than the cut-off established by ROC analysis (as designated by our defined threshold docking score (Fig. 2)).

A post-docking optimization protocol, which employs an automated all atom MD simulated annealing energy minimization, with explicit solvation (referred to as SAEM^28^) followed by local docking, which is described in detail in Hengel et al.,^24^ was used in affinity rank ordering the docked poses. The resulting top five virtual hits were identified, and these natural products were purchased in pure form (>95% purity) from AnalytiCon Discovery GmbH (Potsdam, Germany), and experimentally evaluated for binding to GR via surface plasmon resonance (SPR) which yields stoichiometric binding information, and is thus explicitly able to differentiate specific binders from nonspecific effectors, and will be discussed in more detail below. The utility of SPR is additionally important in the context of natural product screening, due to the scarcity and expense of hits. Compounds that were shown to successfully bind to *H. pylori* GR via SPR were then evaluated for their ability to inhibit GR’s enzymatic activity in a coupled-enzyme assay. Four of the five compounds bound to the protein with micromolar affinity and inhibited *H. pylori* GR with varying degrees of potency and are described in detail below. The reader is directed to a detailed description of this workflow, centered around MD and ROC-based selection, in the Methods section.

Finally, we performed a 50 ns MD simulation on one of these novel active allosteric inhibitors (NP-020560, which was discovered from the workflow outlined above) bound to the *H. pylori* GR-D-Glu complex, as well as a similar study on Compound-A bound to the GR-D-Glu complex. Dynamic cross correlation matrix (DCCM) analyses of these systems clearly indicated specific suppression of inter-monomer correlated motions in the inhibited systems. The loss in inter-monomer correlated motions has never been studied in the case of GR and provides a much-needed mechanism of action for allosteric inhibition of *H. pylori* GR by Compound A and NP-020560.

### Identification of the optimal receptor-docking pair via ROC analysis

**Fig. 3:**
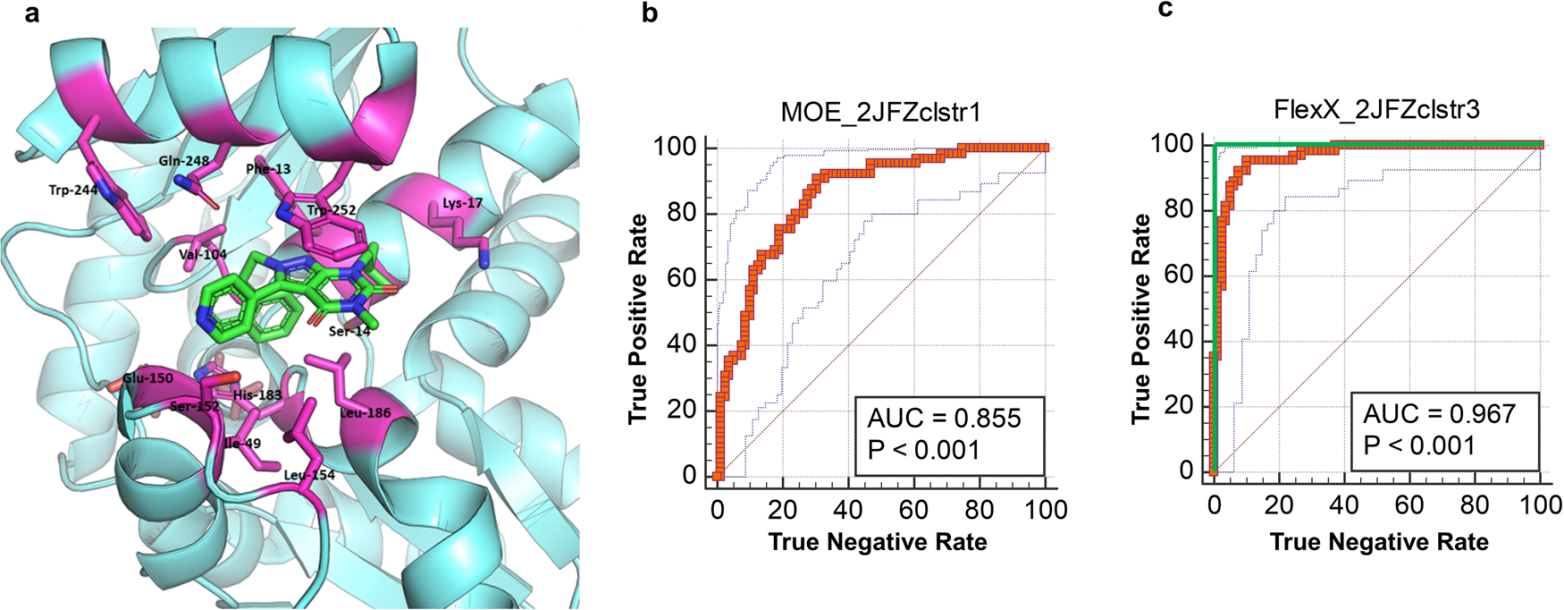
Use of the ROC method for identifying the best docking-receptor pair. Clustering MD snapshots based on allosteric pocket geometries and use of these receptors in the Receiver Operator Characteristic (ROC) statistical procedure for identifying the best combination of receptor and docking protocol for the resolution of true hits and decoys. **a** MD snapshots were clustered based on the structure of the residues in the allosteric pocket (shown in magenta; see Computational Methods for details). 2JFZ provided 6 clusters and 4B1F provided 4 clusters in addition to average and low energy forms for each. **b**, **c** The ROC distributions for the best performing MD cluster for the MOE (**b**) and FlexX (**c**) docking protocols, respectively. The curve measures the ability of the docking procedure to resolve true positives and true negatives. The fraction of true positives that score above a user defined threshold (sensitivity) is plotted on the *y*-axis and the fraction of true negatives (specificity) that score above the threshold is shown on the *x*-axis. The theoretically perfect curve is shown in green, while the average score for both the MOE and FlexX protocols with cluster ‘2JFZclstr1’ and ‘2JFZclstr3’ is shown in red, respectively. The dotted lines show the standard deviation for the fit.

The selection of the appropriate protein receptor structure is of paramount importance when employing structure-based drug discovery approaches. This problem is compounded when working with highly flexible enzymes^29,30^. The use of receptors derived directly from crystal structures raises questions about whether ligands that bind in the same pocket associate with similar protein confirmations in solution. Ultimately, what is most useful in the in silico screening process is identification of receptor forms that can best differentiate between actives and inactives. Using MD simulations to sample additional conformational space and refining receptor selection is a known approach for improving screening outcomes^31–34^. To implement this, we performed all atom classical MD simulations on two available *H. pylori* GR dimer-D-Glu-allosteric inhibitor complexes (PBD ID: 2JFZ^8^ and 4B1F^9^) followed by global structural clustering of MD snapshots to generate different receptors forms (Fig. 3a highlights the allosteric site residues used for clustering). Previous work by Jeremy Smith et al. on a wide range of MD-derived receptors found that there was no a priori characteristics that enabled them to know which snapshots of an ensemble would yield the best receptor for identifying true positives^35^. To address this and to identify a receptor-docking pair capable of enriching for true positives while minimizing false positives and negatives, we utilized a statistical method employing the ‘Receiver Operating Characteristic’ or ROC curves approach^21–23,36^, which is fully described in the Methods section, which relies on the use of decoy compounds generated with the DUD-E application^37^ for use as negatives. Briefly, the compounds were docked, and the final docking scores (kcal/mol) were then used for generating ROC curves which were analyzed based on their area under the curve (AUC) (Fig. 3b, c shows ROC curves of the best performing MOE and FlexX data sets; Table 1 shows the tabulated ROC data). An ideal curve would reach the upper left corner of the graph and have AUC of 1.0 (Fig. 3c – green trace). The analysis also enables the determination of the desired docking score cutoff to use for highest enrichment for true positives while avoiding false positives and negatives (Table 1), which is one of the great advantages of the ROC approach^21–23,36^. In general, all forms of 2JFZ outperformed 4B1F while FlexX outperformed MOE (Table 1). MD cluster 3 for 2JFZ (*2JFZclstr3*) when used for docking using FlexX performed the best with AUC 0.9671 and score cutoff of −20.09 kcal/mol and thus was used for performing the virtual screening (Fig. 3c). Importantly, superimposition of Compound A’s position in the cryptic pocket using this approach was nearly identical to the co-crystallized structure (Supplementary Fig. 2) with an RMSD of 0.883 Å (lower than the crystal structure resolution of 1.86 Å), validating the protocol. Additionally, we could not find any simple pocket metric (such as volume, polarity, or druggability metrics^38,39^), which would predict the ROC performance of any given receptor. In other words, the elucidation of the superior performance of ***2JFZclstr3*** in lead selection (Table 1) compared to other receptor forms must be directly determined by ROC plots. Nevertheless, simple pocket scoring metrics did correctly identify that MD clustered forms of the protein had better predictability (in terms of ROC performance) than crystal structure forms (Table 1), which is highly encouraging.

**Table 1:** Summary of the original protein structures along with the clustered MD forms used for identifying best-performing workflow.

Cluster	Number of models in the cluster	MOE		FlexX		Pocket score
		AUC	Optimum score cutoff	AUC	Optimum score cutoff	
2JFZ-crystal structure	—	0.43	−6.55	0.73	−16.06	1.72
2JFZclstr0	87 of 256	0.48	−8.67	0.88	−19.95	4.64
2JFZclstr1	43 of 256	0.86	−7.37	0.83	−17.76	4.42
2JFZclstr2	19 of 256	0.56	−7.60	0.76	−20.09	1.03
2JFZclstr3	18 of 256	0.64	−7.18	0.97	−20.86	3.95
2JFZclstr4	14 of 256	0.60	−7.42	0.88	−19.28	3.96
2JFZclstr5	13 of 256	0.60	−7.65	0.87	−19.26	2.29
2JFZavg	—	0.705	−7.57	0.85	−19.03	3.64
2JFZlowE	—	0.40	−8.46	0.78	−17.21	0.99
4B1F-crystal structure	—	0.83	−7.40	0.62	−6.74	1.36
4B1Fclstr0	73 of 229	0.72	−7.48	0.68	−15.10	3.57
4B1Fclstr1	36 of 229	0.73	−7.42	0.57	−18.81	3.33
4B1Fclstr2	34 of 229	0.75	−7.33	0.53	−17.13	0.76
4B1Fclstr3	29 of 229	0.67	−7.55	0.68	−18.26	2.92
4B1Favg	—	0.67	−7.66	0.70	−14.34	3.20
4B1FlowE	—	0.69	−7.24	0.45	−24.98	2.32

AUC represents area under the curve in the plot after ROC analysis. Optimum score cutoff indicated the threshold value used in ROC analysis. Pocket score as obtained after performing pocket analysis in Molecular Operating environment.

### In silico screening using the ROC-validated receptor-docking protocol

The AnalytiCon MEGx Natural Products Library was screened using the validated approach for FlexX described above (fully described in Methods). This yielded an initial hit rate of 4.7% (not unusually high for in vitro natural product screens) or 177 compounds that scored above the optimum cutoff value of −20.859 kcal/mol. Since docking programs are optimized to best predict the optimum binding pose for a ligand in complex with a macromolecule, we employed an optimized version of our previously developed computationally expensive Simulated Annealing Energy Minimization (SAEM) docking approach as a secondary refinement screen to rank-order the virtual hits based on predicted affinity (fully described in Methods)^28^. Visual inspection of the top high scoring compounds indicated good shape complementarity between ligand and the allosteric site, π-stacking and/or hydrophobic interactions with Trp-252 (major interaction for Compound A), presence of multiple hydrogen bonding and other hydrophobic interactions with allosteric site residues and lack of steric constraints in the docked poses. Natural product compound selection for experimental testing was based on the SAEM binding score, compound cost (which varies widely in natural product libraries) and availability. Five compounds were selected for experimental evaluation (Table 2 lists these top five compounds with their structures scores).

**Table 2:** Summary of top five identified hits.

Structure	Compound ID	MW	FlexX score (kJ/mol)	AutoDock Vina score (kcal/mol)	SPR (K_d_) (μM)	*H. pylori* GR IC_50_ (μM)
	NP-004604	518.47	−21.43	16.178	170 ± 2	425.3
	NP-020560	538.46	−24.99	15.533	54.7 ± 0.3	6.6 ± 3.1
	NP-008029	432.43	−27.40	15.274	910 ± 10	—
	NP-000205	558.50	−22.50	14.745	80.1 ± 0.6	512.8
	NP-004431	482.44	−25.54	14.498	228 ± 2	705.3
	Compound A	439.52	−23.52	—	80.0 ± 0.5	5.0

Structures of hits, Compound ID, FlexX and SEAM docking score, binding affinity as evaluated by Surface Plasmon Resonance and IC_50_ for enzymatic inhibition of *Hp*GR as evaluated by a coupled-enzyme assay. IC_50_ value for NP-020560 represent mean ± SE, *n* = 3.

### Experimental validation of natural product inhibitor hits from in silico screen and ROC analysis

**Fig. 4:**
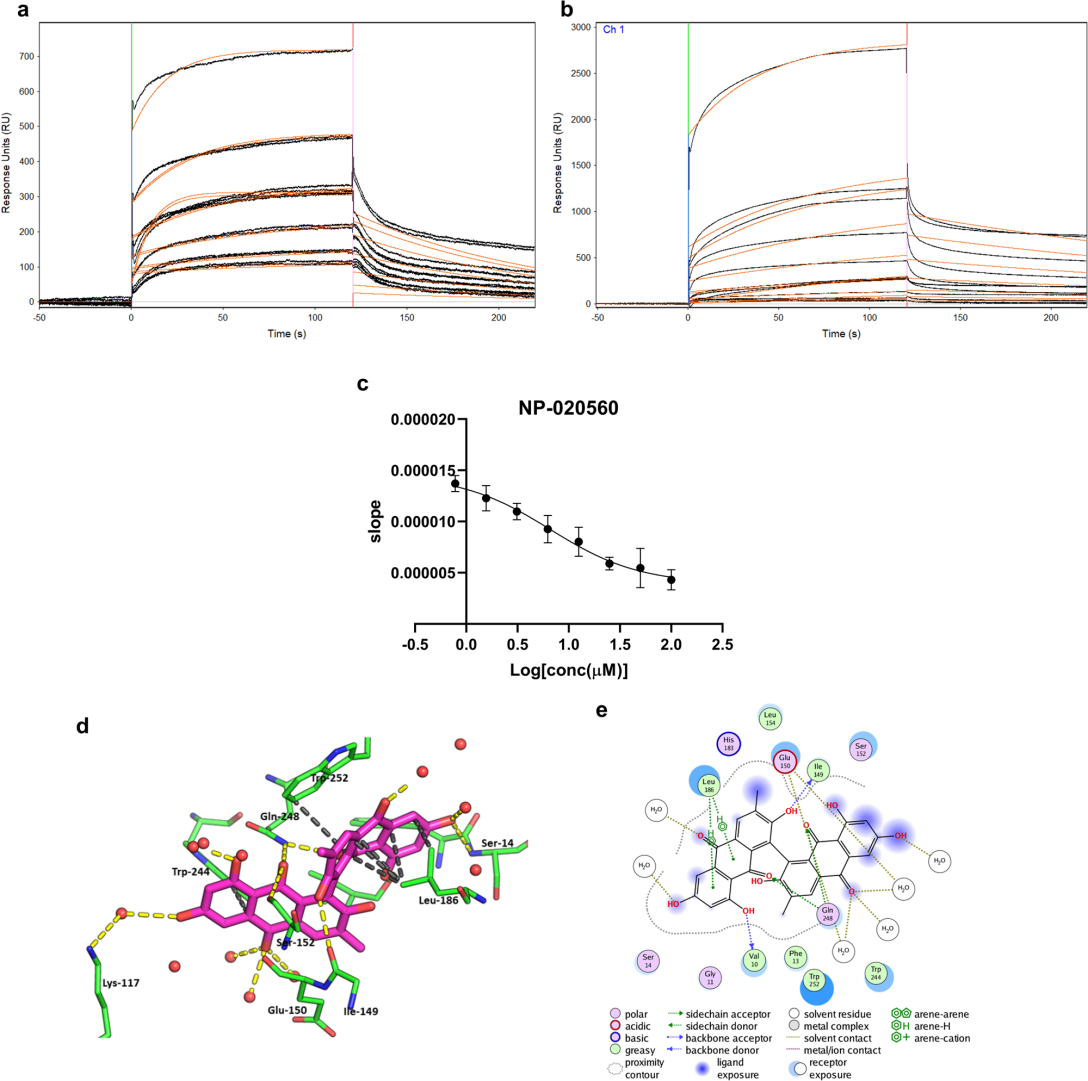
Experimental validation and characterization of the binding of natural product hits obtained from the in-silico screening to *H. pylori* GR. **a**, **b** Compounds were injected onto the sensor in increasing concentration using a dilution series ranging from 500 µM to 7.81 µM (7 concentrations for each natural product hit). Using a Langmuir 1:1 binding model, the *K*_d_ for the samples were obtained as listed in Table 2. Compound A used as control had a *K*_d_ of 80 µM while the two most potent natural product hits NP-020560 and NP-000205 had *K*_d_ of 54.7 ± 0.3 µM and 80 ± 0.6 µM, respectively (**a**, **b**, Table 2). The binding affinity of the rest of the three compounds is as listed in Table 2 and the SPR graphs are as shown in Supplementary Fig. 3. These binding experiments indicate that compound A and our hits from virtual screening bind to *H. pylori* GR stoichiometrically in the absence of the substrate (D-Glu). **c** Evaluation of inhibitory activity of NP-020560 against *H. pylori* GR employing a previously established coupled-enzyme assay^40^. Four of the five tested compounds inhibited the enzyme to different extents with NP-020560 being the most potent, having an IC_50_ of 6.6 ± 3.1 µM (which is roughly equivalent to the IC_50_ for compound A, see Table 2), while the other compounds had IC_50_ values in the high micromolar range (Table 2). Values represent mean ± SE, *n* = 3. **d**, **e** NP-020560 fits into the allosteric pocket making several polar and hydrophobic interactions. One of the anthracenedione ring of the biantracene ring system in NP-020560 is inserted into the inner pocket making the key π-stacking interaction with Trp-252 (a key recognition feature of Compound A as well) along with forming several hydrophobic interactions with the side chain of Leu-186. The second anthracenedione ring forms an edge to face π-stacking interaction with Trp-244. The hydroxyl and ketone moieties on NP-020560 form several hydrogen bonding interactions with several pocket residues including Ser14, Ile-149, Ser-152, and Gln-248.

We first evaluated the binding affinity of these compounds to *H. pylori* GR by Surface Plasmon Resonance (SPR). A combination of amine coupling and HisCapture was used to immobilize the protein on the surface of the SPR biosensor. Compounds were injected onto the sensor in increasing concentrations using a dilution series ranging from 500 µM to 7.81 µM (7 concentrations). Using a Langmuir 1:1 binding model, the *K*_d_ for the virtual hits was obtained as listed in Table 2. Compound A was used as control and had a *K*_d_ of 80 μM while the two most potent natural product hits NP-020560 and NP-000205 had *K*_d_ values of 54.7 ± 0.3 μM and 80.1 ± 0.6 μM, respectively (Fig. 4a, b, Table 2). The binding curves for the evaluated hits and control compound represent stoichiometric 1:1 binding with reasonable on and off rates and overall binding response (RU), and do not indicate any non-specific binding to the protein or chip. The binding affinity of the rest of the three compounds is as listed in Table 2 and the SPR graphs are as shown in Supplementary Fig. 3a–c. These binding experiments indicate that compound A and our hits from virtual screening bind to *H. pylori* GR in the absence of the substrate (D-Glu), which is contrary to the uncompetitive nature of Compound A originally proposed by Lundquist et al.^8^. After establishing that the virtual hits bind to *H. pylori* GR, we next evaluated the ability of these hits to inhibit the enzymatic activity of the enzyme employing a previously established coupled-enzyme assay^8,40^. Four of the five tested compounds inhibited GR to different degrees, with NP-020560 being the most potent, having an IC_50_ of 6.6 ± 3.1 µM (Table 2 and Fig. 4c), while the other compounds had IC_50_ values in the high micromolar range (Table 2 and Supplementary Fig. 3d–f). Interestingly, NP-020560 has an IC_50_ value within statistical error of Compound A, but clearly binds more strongly than Compound A when measured by SPR (Table 2). Additionally, the difference in *K*_d_ (54.7 ± 0.3 μM) and IC_50_ (6.6 ± 3.1 µM) for NP-020560 indicates that it binds tighter to *H. pylori* GR in presence of its substrate (D-Glu), and this trend is similar to one observed for compound A as reported in the literature, in which changes in intrinsic fluorescence were used to determine *K*_d_ values with and without D-Glu.^15^ The source of these disparities is due to the enormous global conformational changes in GR associated with glutamate binding.^15^. The other natural product leads all have about an order of magnitude weaker IC_50_ values than Compound A.

### The consensus pharmacophore from the structures of bound natural product hits

Next, we examined the binding mode of NP-020560 in the allosteric pocket in more detail. As shown in Fig. 4d, e & Supplementary Fig. 4a, NP-020560 occupies the allosteric pocket making several polar and hydrophobic interactions. One of the anthracenedione ring of the biantracene ring system in NP-020560 is inserted into the inner pocket making the key π-stacking interaction with Trp-252 along with forming several hydrophobic interactions with the side chain of Leu-186. The second anthracenedione ring forms an edge to face π-stacking interaction with Trp-244. The hydroxyl and ketone moieties on NP-020560 form several hydrogen bonding interactions with several pocket residues including Ser14, Ile-149, Ser-152, and Gln-248. As shown in Supplementary Fig. 4 the remaining four virtual hits also make similar binding contacts with π-stacking interaction with Trp-252 and several hydrogen bonding interactions including with Gln-248 being the key contacts.

### The salient feature of allosteric inhibition is dampening of a dynamic C-terminal α-helix

**Fig. 5:**
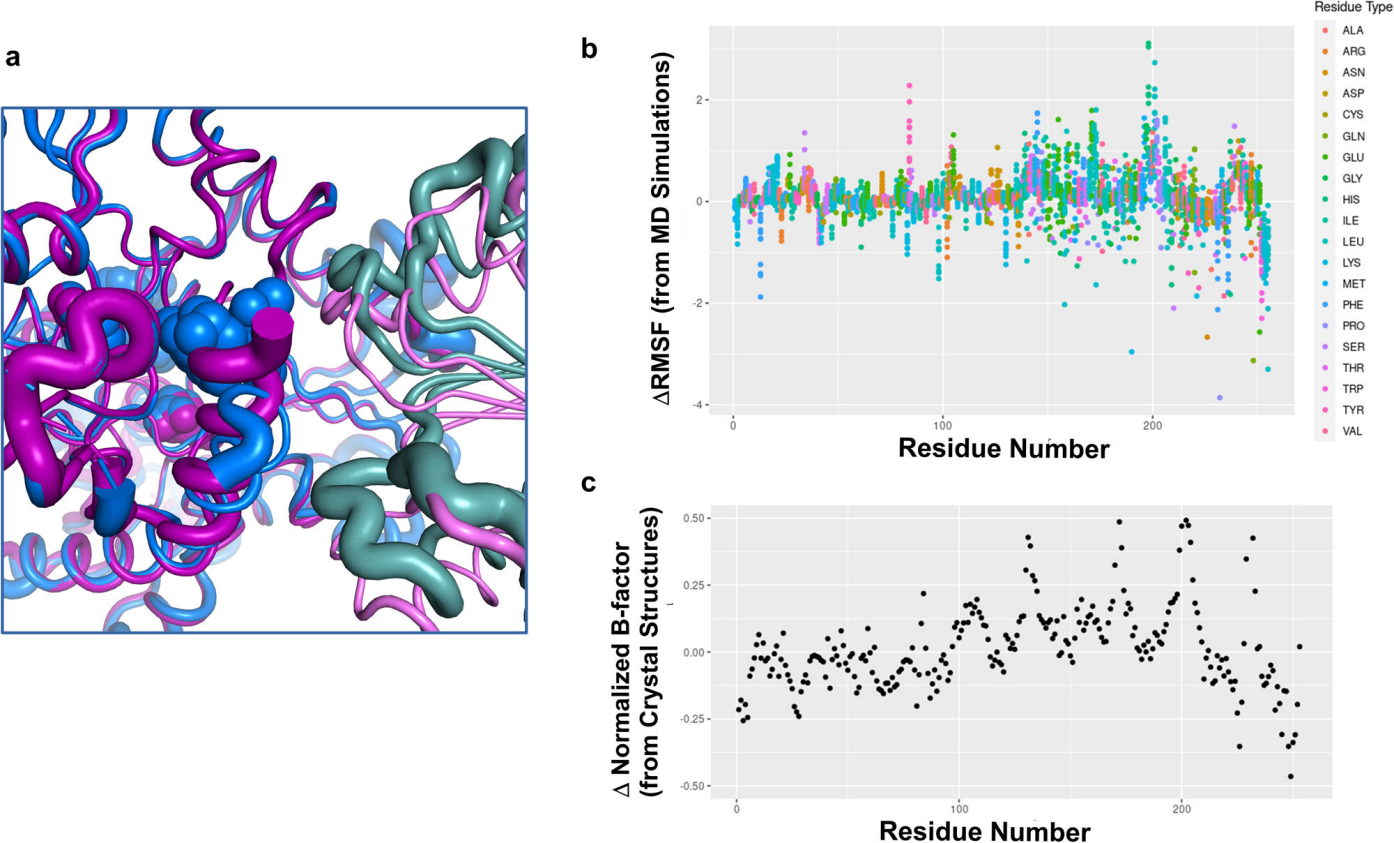
Trends in changes between inhibited and uninhibited GR structures. **a** Heat map of crystallographic B-factors show high B-factor values in the C-terminal α-helix. The left-hand side of the figure shows superpose of the *H. pylori* GR dimer with (2JFZ-blue) and without Compound A (2JFX-purple) bound. The position of Compound A is shown in space filing blue spheres, which highlights the importance of the C-terminal α-helix position in the allosteric site architecture. The half blue and half purple spheres represent D-Glu in both crystal structures. The diameter of the cartoon tubes represents magnitude of the crystallographic B-factor value and the thicker the tubing of the cartoon structure. The C-terminal α-helix of only 2JFX (GR without Compound A bound) has a very large B-factor (wider tube diameter) relative to 2JFZ (Compound A-bound structure). On the right-hand side of the figure, the light purple cartoon structure represents the neighboring unit of 2JFZ and the teal structure is the neighboring unit of 2JFX. An analysis of these neighboring asymmetric units shows no clashes that could account for these selective large differences in B-factors in the C-terminal region, suggesting that dynamics may be an important factor. **b**, **c** A juxtaposition between computational (MD simulations) and experimental (X-ray crystallography) data. In panel **b**, the changes in RMSF (Å) between the inhibitor-bound system (**GR-D-Glu-NP-020560**) and the inhibitor-free system (**GR-D-Glu**) are shown for MD simulations as a function of the residue number. In **c**, the changes in normalized B-factors between the inhibitor-bound structure (**2JFZ**: GR-D-Glu-Compound-A) and the inhibitor-free system (**2JFX**: GR-D-Glu) are plotted as a function of residue number. In both cases, **b** and **c**, the data are derived from subtracting the uninhibited data from the inhibited data (Supplementary Fig. 6).

A superimposition of structures 2JFX (*H. pylori* GR-D-Glu) and 2JFZ (*H. pylori* GR-D-Glu-Compound A) shows very few distinct differences, with a notable exception around the C-terminal helix, which can be seen to significantly move in order to accommodate the allosteric inhibitor. However, Fig. 5a also shows that there is a very large increase in the B-factors for the C-terminal region, which does not appear to be connected with crowding from the neighboring units. These observations suggests that there may be useful dynamical information contained within the changes in the normalized B-factors between these two data sets, which has heretofore not been addressed.

Having identified the above natural product allosteric inhibitors of *H. pylori* GR provided us with a unique opportunity to further probe this mechanism of allosteric inhibition, in addition to that of complexation with Compound A. An all-atom MD simulation was performed on 2JFX (uninhibited GR, bound to D-Glu), 2JFZclstr3 (GR inhibited allosterically by Compound A, bound to D-Glu) and GR allosterically inhibited by NP-020560 (from 2JFZclstr3, as described above, also bound to D-Glu). The simulations were performed using the AMBER14 force field in YASARA Biosciences software package for 50 ns with snapshots being collected every 100 ps (see Methods section for simulation details)^41–43^. In Fig. 5b, c we compare the ΔRMSF per residue (inhibited minus uninhibited, using NP-020560), obtained from the MD simulations juxtaposed with the Δnormalized B-factors per residue (inhibited minus uninhibited, 2JFZ-2JFX). The juxtaposition between the two plots is striking; there is a clear similarity in the major peaks and troughs between the MD and X-ray crystallography data. Notably, there is a large trough in the C-terminal α-helical region, signifying a loss of motion in this region in both the MD and crystallography data upon binding the allosteric inhibitor. Additionally, comparison of panels b and c, shows large peaks centered around residue 200 and 171, for both MD and X-ray crystallography data, which indicates two small loop regions that are positioned at the opposite end of α-helices 156–168 and 187–199, which point into the allosteric pocket. Extraordinarily, we see that changes in B-factors, due to binding of an allosteric inhibitor can be directly traced to dynamical changes in the enzyme as calculated from MD simulations of these systems. The ΔRMSF per residue was also calculated from the MD simulations with Compound A (Supplementary Fig. 5a), which, not surprisingly, yields the same pattern as with NP-020560. Additionally, the ΔRMSF per residue (for both NP-020560, Supplementary Fig. 5b, as well as Compound A, Supplementary Fig. 5c) and Δnormalized B-factors per residue (for Compound A, Supplementary Fig. 5d) for the B-monomer exhibits the same pattern, albeit with a lower signal-to-noise ratio, which is an asymmetry in the dimer that is more fully evaluated in the DCCM analysis discussed below. Overall, these data provide an extraordinary structural nexus between atomistic simulations and experimental data, due to the action of allosteric enzyme inhibition, which is the foundation for the deeper insights from observed changes in correlated motion, *vide infra*.

### Occupancy of the allosteric site by either NP-020560 or Compound A yields non-optimal Cα proton transfer geometries

**Fig. 6:**
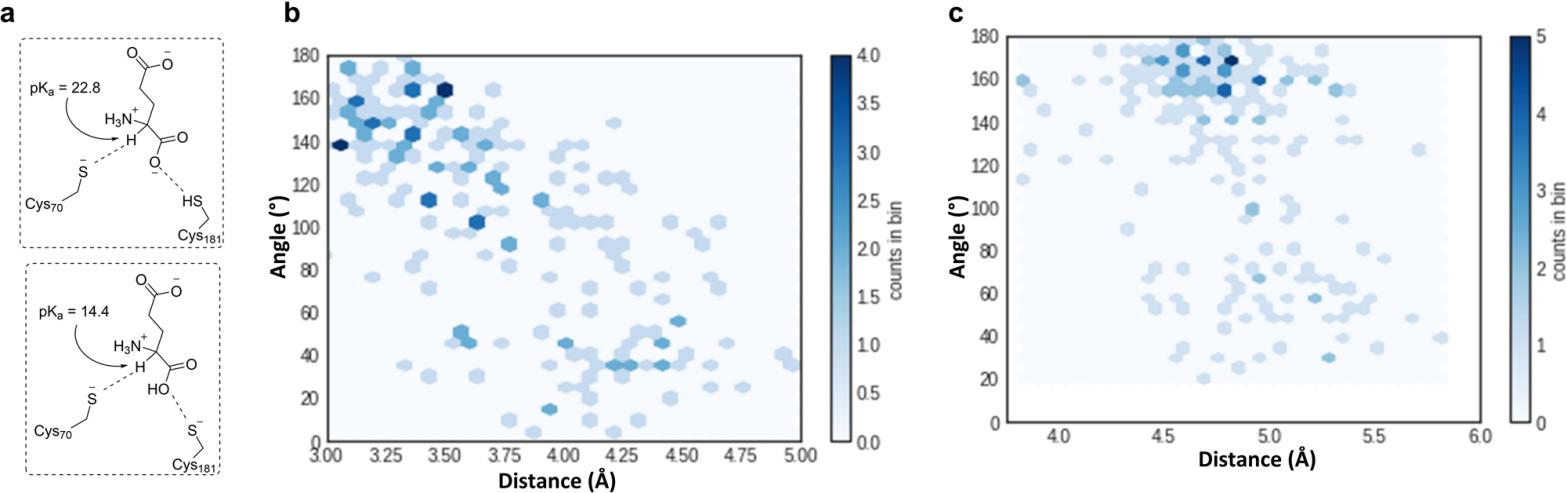
The structural consequences of complexation with NP-020560 on *H. pylori* GR dynamics. **a** Acidification of Cα carbon of glutamate substrate due to protonation of Cα-carboxylate oxygen by catalytic Cys-181. This has been shown in previous studies to be an important dynamical element in *H. pylori* GR. **b** Plot of near attack geometries for Cα deprotonation for MD simulation with *H. pylori* GR bound D-Glu (Cys-S-----H---Cα angle, and Cys-S------Cα distance). **c** Plot of near attack geometries for Cα deprotonation for MD simulation with *H. pylori* GR bound to NP-020560 and D-Glu (Cys-S-----H---Ca angle, and Cys-S------Cα distance).

Glutamate Racemase performs an exotic cofactor-independent “two base” (Cys181 and Cys70) racemization reaction to catalyze the stereo-inversion of glutamate^15,44,45^. Computational studies using MD/QM/MM have found that there is a Cys181-mediated “pre-activation” step, which enables racemization of D-Glu by GR, in the absence of any cofactors such as chelating metals or PLP (a remarkable chemical feat)^15^. Cys181 (one of the two flanking bases) donates a proton to α-carboxylate oxygen of the D-Glu substrate; model studies show this change in substrate protonation state lowers the pKa of its α-carboxylate carbon from 22.8 (carboxylate form) to 14.4 (carboxylic acid form) (Fig. 6a), dramatically reducing the overall barrier for Cα proton transfer (the difficult and rate determining step in amino acid racemization). This chemical pre-activation is linked to larger global allosteric motions in the *H. pylori* GR dimer, which remain, heretofore, undefined. The use of MD simulations with both the newly discovered natural product inhibitor, as well as Compound A, have shed significant light on how such an allosteric path is occurring, which is defined below.

In terms of the effects of these dynamics on active site catalytic chemistry, the orientation and distance of Cys181 from the α-carboxylate oxygen of D-Glu is important for the for the pre-activation step (i.e., protonation of the Cα-carboxylate oxygen). Only after the proton transfer to the α-carboxylate oxygen does the pKa of C-α hydrogen reduce enough to facilitate its abstraction by Cys70^15^. During MD simulation of *H. pylori* GR inhibited by NP-020560, Cys181 rotates frequently between conformations facing towards and away from D-Glu (Fig. 6b), relative to the uninhibited simulation. More importantly, the distance between Cys181 and α-carboxylate of D-Glu is considerably higher in the presence of NP-020560 as compared to uninhibited state of GR (Fig. 6c, d), hindering pre-activation chemistry. Overall, what we observe is that when NP-020560 is bound in the allosteric pocket, it alters the distance and orientation of Cys181 (part of the active site) and predisposes the enzyme to a conformation that is unfavorable to perform the racemization.

### Determination of the source of the disruption in allosteric communication by small molecule inhibitors of *H. pylori* GR

**Fig. 7:**
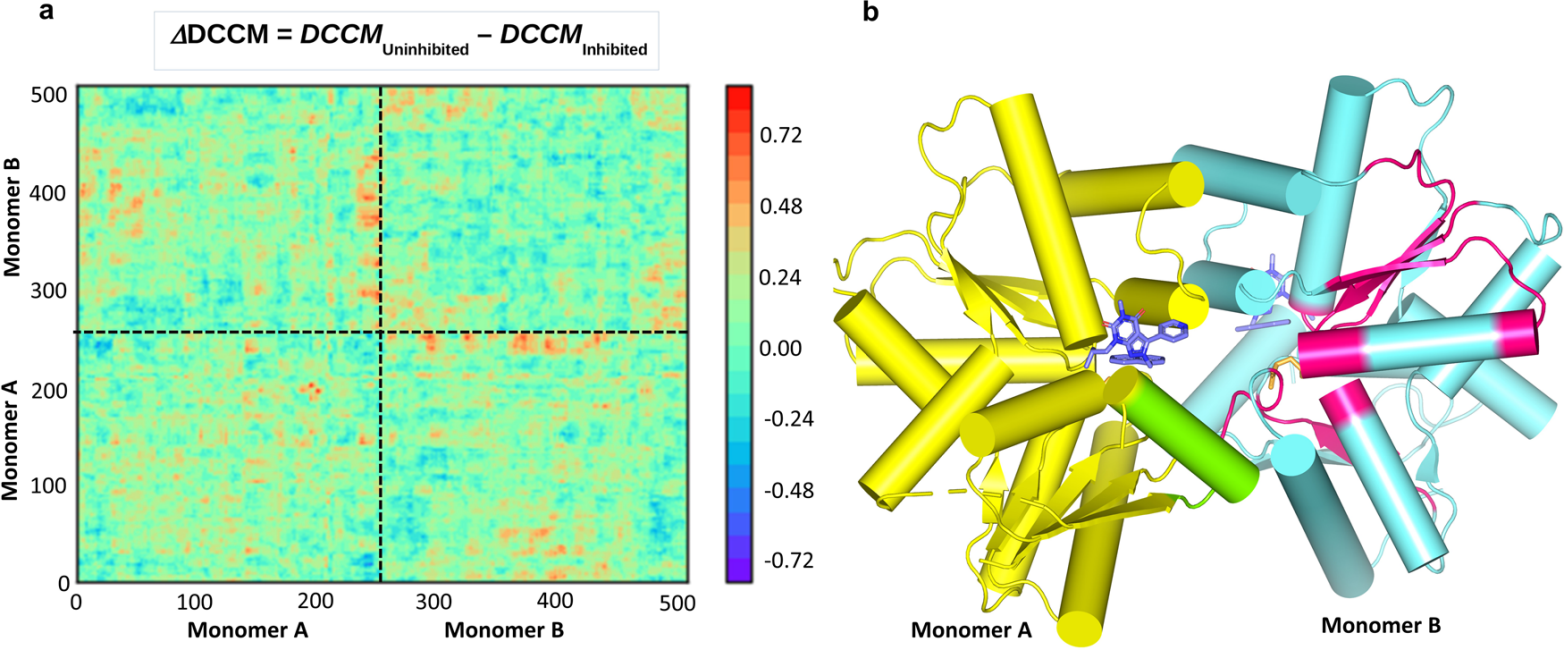
Topology map of the difference between the DCCM for the uninhibited system (GR-D-Glu) and the inhibited system (GR-D-Glu-NP-020560). ΔDCCM = DCCM-_Uninhibited_ – DCCM_Inhibited_. The original DCCM plots are shown in Supplementary Fig. 8. Panel **a** shows that most coupled motions are not different between the two systems, except for a strong region of positive changes in coupled motion are seen at precisely the interaction of subunit A’s C-terminal α-helix (residues 235–255) and a number of structural elements of subunit B; this salient loss of coupled motion is represented in the striking “L-shaped” pattern, which corresponds to an interaction *between* monomers, which is surprising and unprecedented in the GR enzyme family. The affected areas include large portions of the active site, which encompasses the catalytic Cys181, which is responsible for the substrate acidification. Panel **b** shows this key coupled motion, which is lost upon complexation with the allosteric inhibitor, mapped onto the dimeric structure, in which the C-terminal α-helix is shown in green and the regions it is coupled to are shown in red.

To further understand the effect of occupation of the allosteric pocket on the overall dynamics of GR, a dynamic cross-correlation matrix (DCCM) analysis was performed on various complexes of *H. pylori* GR. DCCM provides information on dynamic relationships between all residues of the protein shedding light on how each residue’s motion correlates with every other residue during a MD simulation. (A full description of this technique is located in the Methods section, and a comprehensive application to GR can be found in Dean et al.^46^). To explore the effect of NP-020560 on the entire GR dimer, the DCCM for GR-NP-020560-D-Glu was subtracted from the DCCM of native GR-D-Glu. This provided a difference DCCM (ΔDCCM) as shown in Fig. 7a and it highlights the coupled motions that are lost (or gained) in the presence of NP-020560. The color bar of the topographical map in Fig. 7a indicates the intensity of the loss in coupled motion due to the binding of the NP-020560 inhibitor, with red being the greatest loss and blue being a gain in coupled motion. As we can see, most of the topography is very small and close to zero, which brings the important structural dynamics into stark relief. Indeed, the ΔDCCM is particularly insightful for our purposes of trying to define a set of allosteric structure activity relationships (ASAR) for this difficult to understand inhibition mechanism. The salient loss of coupled motion is represented in the striking “L-shaped” pattern, which corresponds to an interaction between monomers, which can be pinpointed to the C-terminal α-helix (Fig. 7a). Specifically, the C-terminal residue of monomer A (residues 235–255) loses coupled motion with several key helices and sheets in monomer B (Fig. 7a, b). Again, the C-terminal α-helix composes nearly half of the residues making up the allosteric site with Trp252 being the key residue that forms π-stacking interactions with both NP-020560 and Compound A. Importantly, this same pattern also appears in the ΔDCCM for Compound A (Supplementary Fig. S7).

Based on the previous and current observations, it is clear that the pre-activation step (protonation of the Cα carboxylate) is key to the ability of GR to carryout stereo-inversion of D-Glu and is dependent on the highly flexible and dynamic nature of GR. Occupancy of the cryptic allosteric pocket by a small molecule dampens coupled motions *between* two monomers of GR (and this is the operative mechanism for Compound A as well). This reveals the existence of a deeper monomer-monomer interaction in GR in the uninhibited state, the inhibition of which is a novel means of allosteric control by a small molecule, and for the first time explains why GR enzymes in general are almost always found as active dimers. Indeed, this supports the possibility that *H. pylori* GR must be a viable dimer to have catalytic activity. This fundamental knowledge about how *H. pylori* GR is harnessing catalytic power via monomer-monomer cross-talk is a very unexpected and important result from this allosteric drug discovery campaign.

## Discussion

*Helicobacter pylori* glutamate racemase is an essential protein for microbial survival and is an attractive drug target. Due to its high inherit flexibility and poor druggability at the active site, application of classic structure-based design approaches has been challenging. These challenges were overcome by developing a hybrid molecular dynamics-based docking workflow, which targeted a cryptic allosteric pocket, taking into account both global dynamics in the enzyme as well as the fitness of the receptor for selecting true positives in in silico screening. The key steps in the protocol include an all-atom MD simulation on the starting protein to evaluate additional conformations followed by using a decoy library to challenge the workflow and identify the best performing receptor-docking pair for virtual screening. The power of the developed workflow was demonstrated by screening and identifying natural product inhibitors of *H. pylori* GR.

Additionally, molecular dynamics studies with and without the allosteric inhibitor yielded a striking comparison to changes in the normalized B-factors from the crystallographic data, strongly pointing to dynamics as the source of these differences. Analyses of these MD simulations uncovered the existence of monomer–monomer interactions that are dampened by the occupancy of NP-020560 (or Compound A) in the cryptic allosteric pocket. This further reveals a novel mechanism of allosteric control which works by dampening the coupled motions of several key helices within and between monomers. The outcome is a reduction in the flexibility of GR which in turn also orients Cys181 in an unfavorable orientation to carry out the key pre-activation step of Cα-carboxylate protonation, thus inhibiting the ability of GR to stereo-invert D-Glu. Importantly, these studies suggest that the native (i.e., uninhibited) dimeric GR relies on a complex monomer–monomer allosteric crosstalk for its catalytic activity. These studies provide a foundation for an allosteric-centered structure activity relationship for targeting *H. pylori* GR, and a rationale for why GR enzymes largely operate as obligate dimers.

## Methods

### Clustering of receptors

We performed all atom classical MD simulations on two available *H. pylori* GR dimer-D-Glu-allosteric inhibitor complexes (PDB ID: 2JFZ^8^ and 4B1F^9^) using the YAMBER3 knowledge-based force field in YASARA biosciences^41–43^. We chose the two protein complexes because of differences in resolution (4B1F = 2.05 Å; 2JFZ = 1.86 Å) and the presence of structurally analogous inhibitors with different affinities for GR. 2JFZ lacked a loop which was build using the homology model functionality in YASARA Structure package from YASARA biosciences^42^. Before starting the MD simulations, the starting structures were energy minimized followed by generation of periodic simulation cell with explicit solvent extending 10 Å from the surface of complexes. The simulation cell was then neutralized with NaCl (0.9% by mass) as described previously^47^. The MD runs were performed in YASARA following methods as described in Dean et al.^46^, with minor modifications. The simulations were performed using YAMBER03 force field^41^ that uses long-range Coulomb electrostatic potentials calculated using Particle Mesh Ewald^48^ method with a van der walls cutoff of 7.86 Å. The calculations were ran using NVT ensemble at a temperature of 25 °C and pH 7.4 for a total of 700 ns for 2JFZ and 120 ns for 4B1F with snapshots collected every 100 ps. The root-mean-square deviation (RMSD) of the structures was performed using YASARA MUSTANG8 from YASARA Biosciences, while the MD trajectories were analyzed using MDAnalysis toolkit^42^. Lastly, the MD snapshots produced by YASARA were clustered using the Ensemble Cluster tool of the UCSF Chimera package^49^ which applies the methodology of Kelley et al.^50^ (developed by the Resources for Biocomputing, Visualization, and Informatics at the University of California, San Francisco and supported by NIGMS P41-GM103311). The ensembles achieved global RMSD convergence at 60 ns for 2JFZ (Supplementary Fig. 9a) and at 15 ns for 4B1F (Supplementary Fig. 9b) and trajectories from the equilibrated portion of the MD run were clustered based on cryptic allosteric site residues: Val10, Gly11, Phe13, Ser14, Lys17, Ile149, Glu150, Ser152, Leu154, His183, Leu186, Trp244, Gln248, Trp252, and Leu253. These residues were all within 5 Å from compound A in 2JFZ (Fig. 3a). The resulting centroids from clusters representing 75% of MD trajectories (Table 1) along with the time-averaged, low energy and PDB deposited structures of both receptors were used for further studies.

### Generation and preparation of known actives and decoys ‘test’ compound library

A library of known inhibitors (actives) and decoys was generated to test the ability of the workflow to correctly identify true positives and minimize false positives and false negatives. A library of 65 active pyrazolopyridiminedione analogs was compiled from published AstraZenaca studies and respective inactive/decoy compounds were generated using the Database of Useful Decoys – Enhanced (DUD–E) website (Supplementary Table 1)^37^. In short, decoys are property-matched to true actives using physicochemical properties like molecular weight, logP, number of hydrogen bond donors and acceptors, etc. but are distinct in chemical topology to true binders. On average, one decoy was retained for each active compound to give a library of 144 compounds (Supplementary Table 1) that was preprocessed in Molecular Operating Environment (MOE) program by adding hydrogens, adjusting partial charges and energy minimizing to give the final library used for the study^38^.

### Docking known actives and decoys ‘test’ compound library

Two different docking programs where then used since each takes a different approach for ligand placement and for searching minimum energy confirmations: FlexX (part of LeadIT available from BioSolveIT GmbH)^51,52^ and MOE 2016^38^. The 144-compound test library was docked into the cryptic allosteric pocket of each of the 16 protein structures (9 for 2JFZ and 7 for 4B1F) (Table 1).

For MOE, the site for docking was defined by selecting the protein residues within 5 Å from co-crystallized ligand. Docking parameters were set as Placement: Triangle matcher; Scoring function: London dG; Retain Poses: 30; Refinement: MMFF94x force field based refinement; Re-scoring function: MM/GBSA dG (this includes an implicit solvation energy calculation and captures changes in the solvent exposed surface area of the pose, and is a highly parameterized version of the popular MM/PBSA and MM/GBSA methodologies^53,54^; Retain poses: 1.

FlexX is a module available within the LeadIT software package and it predicts protein–ligand complexes by fragmenting a ligand at rotatable bonds, determining and docking a base fragment, and incrementally building up the ligand^51,52^. For docking, individual protein were loaded into FlexX and the binding site was defined by selecting the protein residues within 5 Å from co-crystallized ligand (Compound A). Docking parameters were set at default with base placement employing the hybrid approach and retention of 1 pose per compound. The program performed well with the best pose for Compound A having rmsd of 0.883 Å

### Receiver Operator Characteristic (ROC) using decoys

To identify the best docking protocol capable of enriching for true positives and reduce false positives and false negatives we used a statistical method employing ROC curves^21^. For generation of the curves, all ‘actives’ were assigned the value of 1, while all decoys were named as 0. The corresponding docking score for each compound for a receptor-software pair was noted. ROC curves were then created using MedCalc program for each of the receptor-software pair by scoring ranks of actives vs inactive poses^55^. The generated plot represents ‘False positive rate’ on the *x*-axis and ‘True positive rate’ on the *y*-axis for a wide range of cut-off scores, and helps examine how well a classifier (i.e., the cutoff threshold chosen for a particular scoring function) is capable of correctly selecting actives and discarding inactives (Fig. 3b, c, Table 1). The curves were analyzed using the metric of the area under the curves (AUC)^22^. An ideal curve would reach the upper left corner of the graph, while a random classifier would cross the diagonal of the graph area (Fig. 3c). The best performing receptor-software pair had curves reaching close to ideal curve with an AUC close to 1.

### Rationale for library selection

Natural product libraries are often the best place to begin a screening campaign, having a storied history in drug discovery. However, the expense of isolation, purification, and structural validation have often outweighed their advantages. In silico screening from commercially available collections of natural products opens up new possibilities for structure-based drug discovery. Although employing a natural product library can be about an order of magnitude more expensive than traditional libraries, the higher hit rate and distinct chemical space of natural products may prove advantageous for highly benchmarked workflows such as the one presented here. To challenge our protocol, we screened a library of natural products from AnalytiCon Gmbh (Potsdam, Germany) (MEGx Purified Natural Product Screening Compounds), which is a collection of 3734 compounds.

### In silico screening

An in silico version of the AnalytiCon Discovery MEGx Natural Products Screen Library along with Compound A as control was prepared for screening as described above and docked into the cryptic allosteric pocket of 2JFZclstr3 (best performing receptor) following the procedure detailed above using FlexX (best performing docking program). The top scoring pose for each of the 177 compounds scoring above the score cutoff of −20.859 kcal/mol were exported as individual pdb files to be used for SEAM-docking.

### All atom-simulated annealing energy minimization (SAEM) – docking protocol

With FlexX being optimized to identify true binders, we decided to employ the SAEM approach^23,28^ as a secondary screen, which is designed to provide reliable rank-ordering of the hits. The all atom simulated annealing energy minimization with YAMBER03 knowledge-based force field followed by rescoring in AutoDock Vina^56^ is a customized program completely automated with a script in the Python-based Yanaconda scripting language and run in the YASARA software package^41,42^. We employed a slightly modified version of the method explained in detail in our previous reports^28,47^. In short, each chosen complex (output from FlexX docking) was placed in a simulation cell, solvated, charge-neutralized followed by optimization of solvent and hydrogen bonding network and then phased simulated annealing was performed without any restraints as described in Whalen et al.^28^. All the protein and ligand atoms were kept free. The convergence criteria for the run was reaching the energy minimum with a maximum of five failures allowed. Once the convergence was reached, the affinity of the ligand in this optimized complex along with binding energy was determined by employing the docking utility of AutoDock Vina^56^ and scoring with the same. A key step in this process is the retention of interstitial water molecules during the AutoDock Vina docking and scoring.

### Procedure for DCCM and ΔDCCM

The DCCM between residues i and j was calculated by dividing the dot product of any two residue displacements relative to an average structure, as described by Equation 1:

$$DCCM_{i,j} = \frac{{\left\langle {\overrightarrow d _i \cdot \overrightarrow d _j} \right\rangle }}{{\sqrt {\langle {d_i^2} \rangle \langle {d_j^2} \rangle } }}$$

The value of *d* is the displacement of an atomic position from the ensemble average position, and the brackets represent averaging over the snapshots from the MD simulation of the *H. pylori* GR-ligand complexes. The ΔDCCM values were calculated using the Pandas package within Jupyter Notebook Release 6.1.4

### Expression and purification of *H. pylori* glutamate racemase

*E. coli* BL21 (DE3) pLysS cells were transfected with 6XHis-tagged H. pylori glutamate racemase (GR) and GroEL/ES chaperone proteins inserted in the pET-15b and pCH1 vectors, respectively. Cells were cultured overnight at 37 °C with rotation in 5 ml of Terrific Broth (TB), supplemented with 50 μg/mL ampicillin, 30 μg/mL chloramphenicol, and 100 μg/mL kanamycin. The 5 mL starter culture was back‐diluted into 750 mL of TB medium containing antibiotics, and grown at 37 °C with shaking until the OD600 reached 0.8–1.0. Protein expression was induced upon addition of IPTG at a final concentration of 0.1 mM. Following induction, protein was expressed for 16–18 h at 20 °C with shaking. Cells were harvested by centrifugation at 5000 × *g* at 4 °C for 20 min. Supernatant was discarded and cell pellets were resuspended in buffer A (100 mM Tris, 100 mM NaCl, 10 mM imidazole, 1 mM TCEP, pH 8.0). An Emulsiflex microfluidizer was used to lyse the cells. Insoluble materials were pelleted by centrifugation at 30,000 × *g* for 75 min at 4 °C and the supernatant was passed through a 0.22-μm filter. Clarified lysate containing 6XHis‐tagged protein was then loaded onto a 1 mL HisTrap IMAC HP (GE Healthcare) cobalt resin column equilibrated with buffer A. Cobalt-bound GR was washed with buffer A and eluted over several fractions via a linear gradient from buffer A to buffer B (100 mM Tris, 100 mM NaCl, 250 mM imidazole, 1 mM TCEP, pH 8.0). The purity of select fractions were analyzed using 12% SDS-PAGE gels. Pooled fractions were concentrated utilizing a 10,000 MWCO Amicon centrifugal filter device. Protein destined for crystallography purposes was dialyzed into thrombin cleavage buffer (50 units thrombin, 20 mM Tris, 150 mM NaCl, 25 mM CaCl_2_, pH 8.4) overnight to remove the 6XHis-tag. GR was further purified by Size-exclusion chromatography using a HiLoad 16/200 Superdex column (GE Healthcare) equilibrated with protein storage buffer (50 mM Tris, 100 mM NaCl, 0.2 mM DTT, pH 8.0). Protein stocks were stored at a final concentration of 5–7 mg/mL with 20% v/v glycerol at −20 °C.

### Coupled-enzyme assay for *H. pylori* glutamate racemase

The D- to L-glutamate racemization activities of *H. pylori* GR was assayed through a previously established coupled-enzyme method, utilizing L-glutamate dehydrogenase and diaphorase^40^. The *H. pylori* GR required for this assay was purified as detailed above. The assay consists of 1.25 mM D-Glu, 2.51 mM ADP, 5 mM NAD, 0.506 mM INT, 5 Units of L-GDH, 0.4 units of diaphorase and 2 µM GR in 50 mM TRIS, pH = 8. Compound stocks solutions of varying concentrations (100 µM–50 mM) were made in DMSO and introduced in the assay vials. The final DMSO concentration after addition of compounds in the assay was 5% v/v. The assays were performed as three independent repeats for Compound A and NP-020560, while other hits being weak inhibitors and restricted by amounts available, were evaluated in a single repeat. Absorption for the reduced iodonitrotetrazolium was collected by measuring absorbance at 500 nm at 1 min interval for 60 min on Cary 300 UV-VIS Spectrophotometer from Varian (Palo Alto, CA). Absorbance for each compound concentration at every time point were normalized to absorbance for blank, followed by calculating slopes for each concentration. The calculated slopes with the respective log (inhibitor concertation) were fitted to log(inhibitor) vs response (three parameters) model within ‘Nonlinear Regression-Dose Response Inhibition’ in GraphPad Prism version 8.4.3. The program provides output as IC_50_ and LogIC_50_ along with respective standard errors, and for ease of expression, we report inhibition data as IC_50_ (mean ± SE).

### Surface Plasmon Resonance (SPR) GR immobilization

Experiments were performed using a SensiQ Pioneer SPR. The biosensor chip was first equilibrated with SPR running buffer (50 mM Tris-HCl, 100 mM NaCl, TCEP, 1% v/v TWEEN, pH 8.0), then the surface of the biosensor chip was seasoned by injecting 200 μL 50 mM NaOH at 40 μL/min, 200 μL 0.1% v/v SDS at 40 μL/min, and 200 μL 10 mM HCl at 40 μL/min in the test and reference biosensor chip flow channels. This process of seasoning was repeated twice. All buffers and solutions were filter sterilized with 0.22-μm buffer filters before using on the SPR instrument. After seasoning, the lines were primed three times with SPR buffer. The biosensor chip was then prepped for protein immobilization by washing the HisCap chip with 200 μL of 400 mM EDTA (pH = 8) injected at 20 μL/min followed by a 15 s dissociation phase. Next 500 μL of 100 mM NiCl_2_ was injected at 40 μL/min followed by a 15 s dissociation phase, then 200 μL of 700 mM imidazole was injected at 40 μL/min followed by a 15 s dissociation phase. To use amine coupling in addition to HisCapture, 500 μL of 0.4 M EDC/0.1 M NHS was injected at 40 μL/min, 325 μL of 180 nM GR is injected at 10 μL/min. Protein was injected across the nickel activated channel until it reached the desired response units (RU) of immobilized protein, approximately 8000–12,000 RU. This density of protein on the biosensor surface is necessary for measuring binding interactions of small molecules. Once achieved, the remaining protein was washed out with SPR buffer and buffer was continuously pumped until the baseline stabilized with a drift of no more than 3 RU min^−1^. This was followed by a manual injection of SPR running buffer at 10 μL/min.

### Surface plasmon resonance

*H. pylori* GR was immobilized to the biosensor using a HisCapture couple method detailed above. Serial two-fold dilutions of compound A, NP-020560, NP-000205, NP-004431, and NP-004604 were prepared in running buffer (100 mM TRIS, pH 8; 0.01% v/v Triton X-100) containing 5% v/v DMSO and injected in the test and reference biosensor chip channels at 50 μL/min, followed by a 100 s dissociation phase at 25 °C. Serial dilutions of each compound were injected in triplicate along with several injections of running buffer in random order. All sensograms were analyzed using the program Qdat (2.6.3.0) and it fitted to a model of simple binding of 1:1 Langmuir binding model for all virtual hits. Qdat calculates the microscopic rate of association (*k*_a_) and dissociation (*k*_d_) and using that also calculates dissociation constant (*K*_d_) for each binder tested.

### Reporting summary

Further information on research design is available in the Nature Research Reporting Summary linked to this article.

### Supplementary information

Supplementary Information

Reporting Summary

## Data Availability

Relevant data are available from the corresponding author on reasonable request.
