# Supplementary material for: Decrypting a cryptic allosteric pocket in H. pylori glutamate racemase
Source: Commun Chem. 2021 Dec 10;4:172. doi: 10.1038/s42004-021-00605-z (PMC9169614; doi:10.1038/s42004-021-00605-z)
Supplement: Supplementary file 1 — Supplementary Information [file 42004_2021_605_MOESM1_ESM.pdf]

Supplementary Information

Decrypting a Cryptic Allosteric Pocket in *H. pylori* Glutamate Racemase

Pratik Rajesh Chheda[1], Grant T. Cooling[1], Sondra F. Dean[1], Jonah Propp[1], Kathryn F. Hobbs[2] and M. Ashley Spies[1,2]*

[1] *Division of Medicinal and Natural Products Chemistry, Department of Pharmaceutical Sciences and Experimental Therapeutics, The University of Iowa, Iowa City, Iowa 52242, United States of America*

[2] *Department of Biochemistry, Carver College of Medicine, The University of Iowa, Iowa City, Iowa 52242, United States of America*

Supplementary Figures:

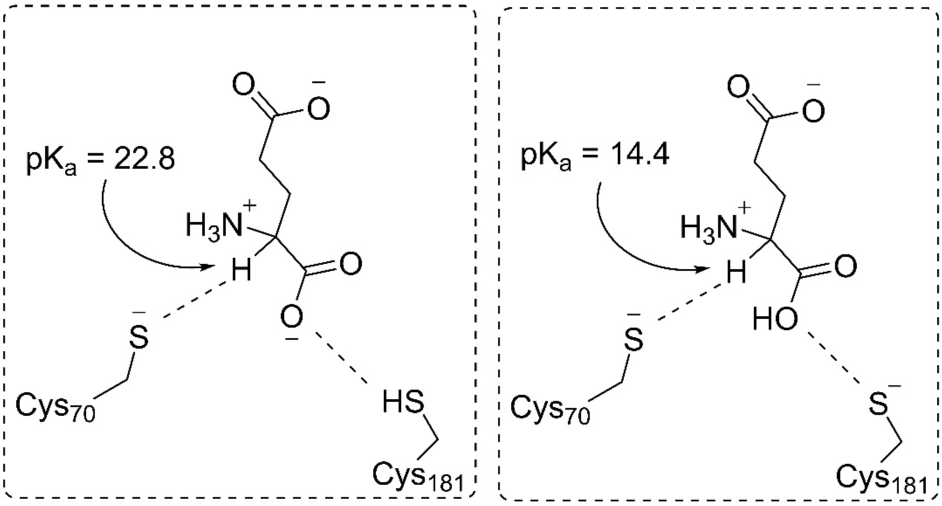

Supplementary Figure 1.

Acidification of Cα carbon of glutamate substrate due to protonation of Cα-carboxylate oxygen by catalytic Cys-181 is necessary to reduce the pK_a and enable stereo-inversion.

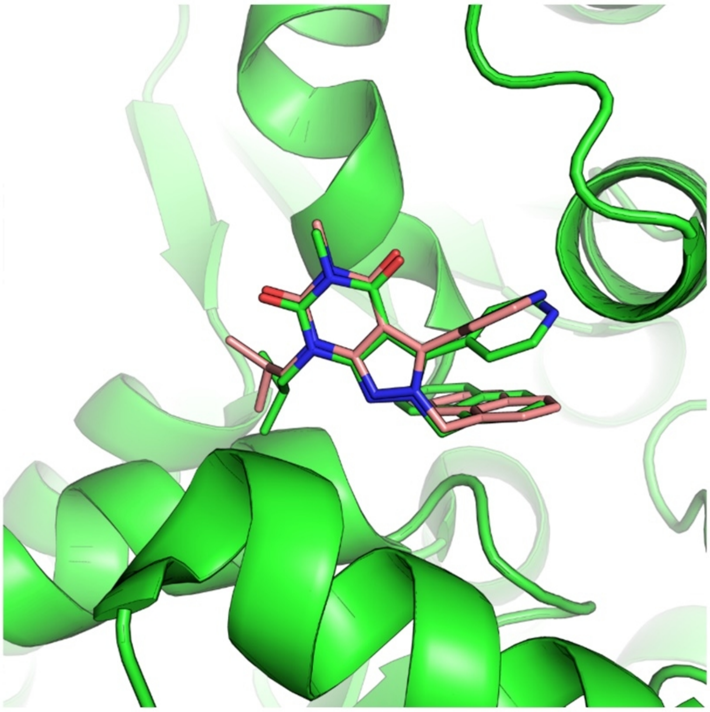

Supplementary Figure 2.

Overlap of compound A pose generated by FlexX onto the co-crystallized ligand in PDB 2JFZ. RMSD of the two poses is 0.883 Å which is lower than the crystal structure resolution of 1.86 Å.

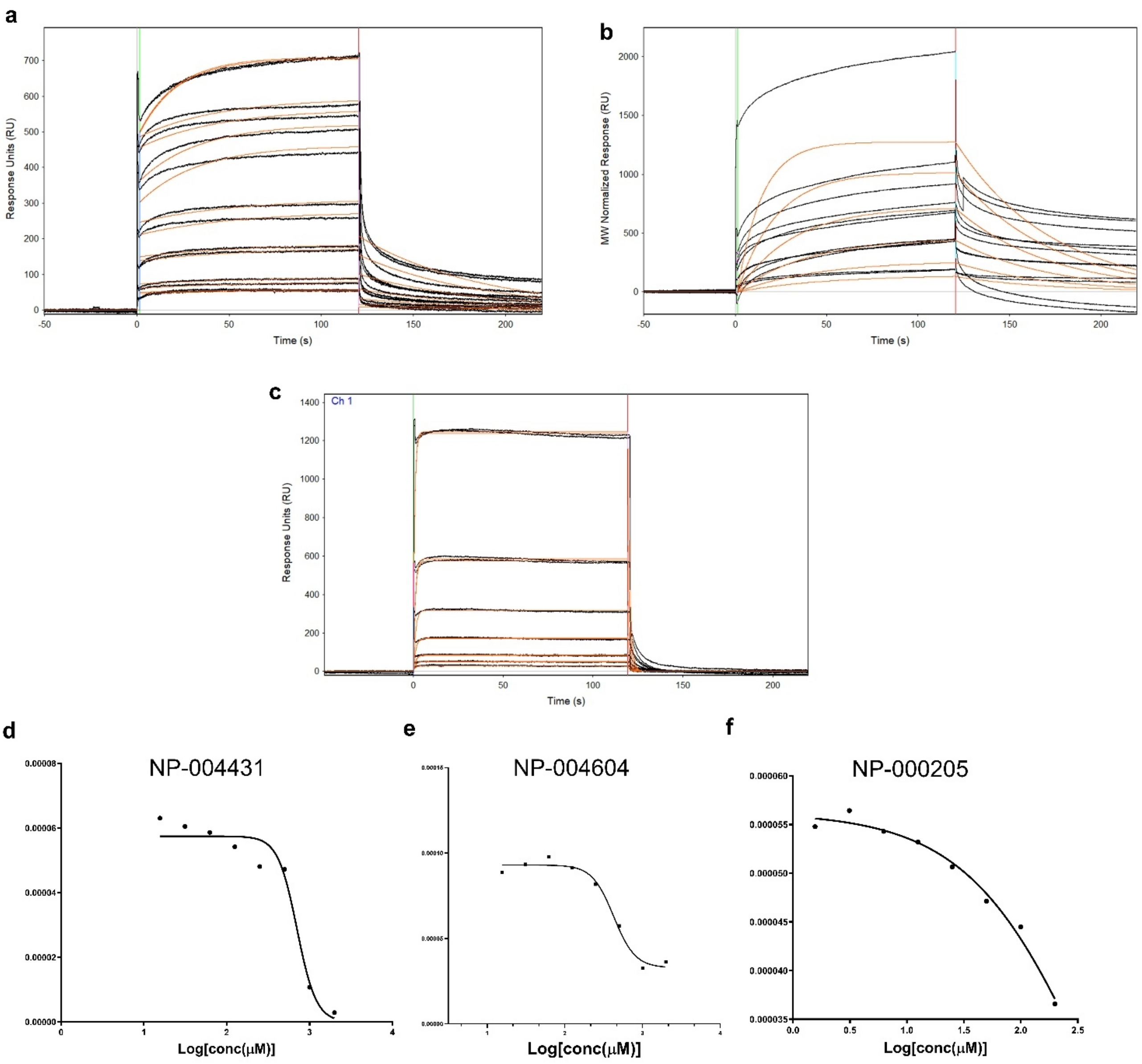

Supplementary Figure 3.

A-C) Representative binding curves of top five hits to glutamate racemase as measured by SPR. Data was fit with a 1:1 binding model shown in orange with raw data shown in black and the K_d was determined by fitting to the same model. Error values represent standard deviation (SD). **A)** Binding curve for NP-004431: measured K_d of 228±2 µM. **B)** Binding curve for NP-004604: measured K_d of 170±2 µM. **C)** Binding curve for NP-008029: measured K_d of 910±10 µM. **D-F)** Representative IC_{50} curves for natural product hits evaluating inhibitory activity of the hits against *H. pylori* GR employing a previously established coupled-enzyme assay[1]. **D)** IC_{50} curve for NP-004431: measured IC_{50} of 705.3

µM. **E)** IC_{50} curve for NP-004604: measured IC_{50} of 425.3 µM. **F)** IC_{50} curve for NP-000205: measured IC_{50} of 512.8 µM.

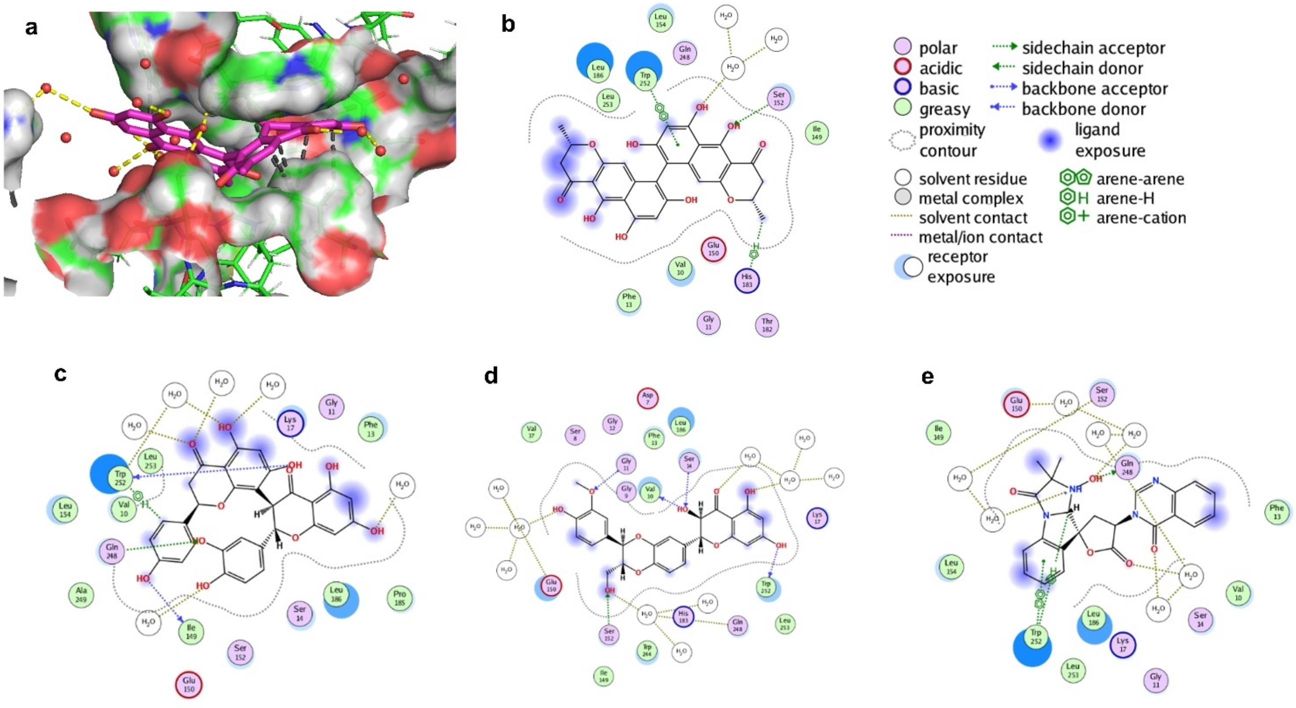

Supplementary Figure 4.

A) Docking pose of NP-020560 occupying the allosteric binding pocket and highlighting key interactions with *H. pylori* GR; NP-020560 is represented as pink sticks and allosteric residues are depicted as green sticks; protein surface showing the occupancy of allosteric site. **B-E)** Ligand Interaction maps for top hits generated in Molecular Operating Environment (MOE) highlighting key ligand-protein interactions with their nature detailed in legend for panel B. **B)** Ligand interaction map for NP004604. **C)** Ligand interaction map for NP000205. **D)** Ligand interaction map for NP004431. **E)** Ligand interaction map for NP008029.

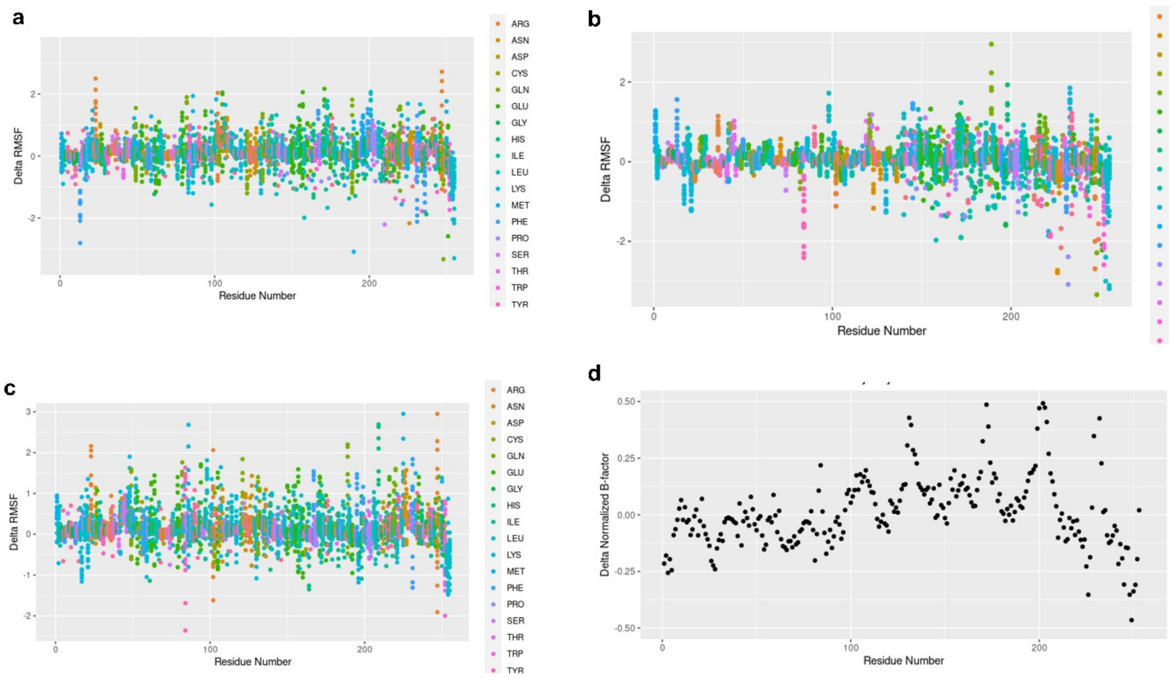

Supplementary Figure 5.

A) The changes in RMSF (Å) between the inhibitor-bound system (**GR-D-Glu-Compound A**) and the inhibitor-free system (**GR-D-Glu**) are shown for MD simulations as a function of the residue number for Monomer A. **B)** The changes in RMSF (Å) between the inhibitor-bound system (**GR-D-Glu-NP-020560**) and the inhibitor-free system (**GR-D-Glu**) are shown for MD simulations as a function of the residue number for Monomer B. **C)** The changes in RMSF (Å) between the inhibitor-bound system (**GR-D-Glu-Compound A**) and the inhibitor-free system (**GR-D-Glu**) are shown for MD simulations as a function of the residue number for Monomer B. **D)** The changes in normalized B-factors between the inhibitor-bound structure (**2JFZ**: GR-D-Glu-Compound-A) and the inhibitor-free system (**2JFX**: GR-D-Glu) are plotted as a function of residue number for Monomer B.

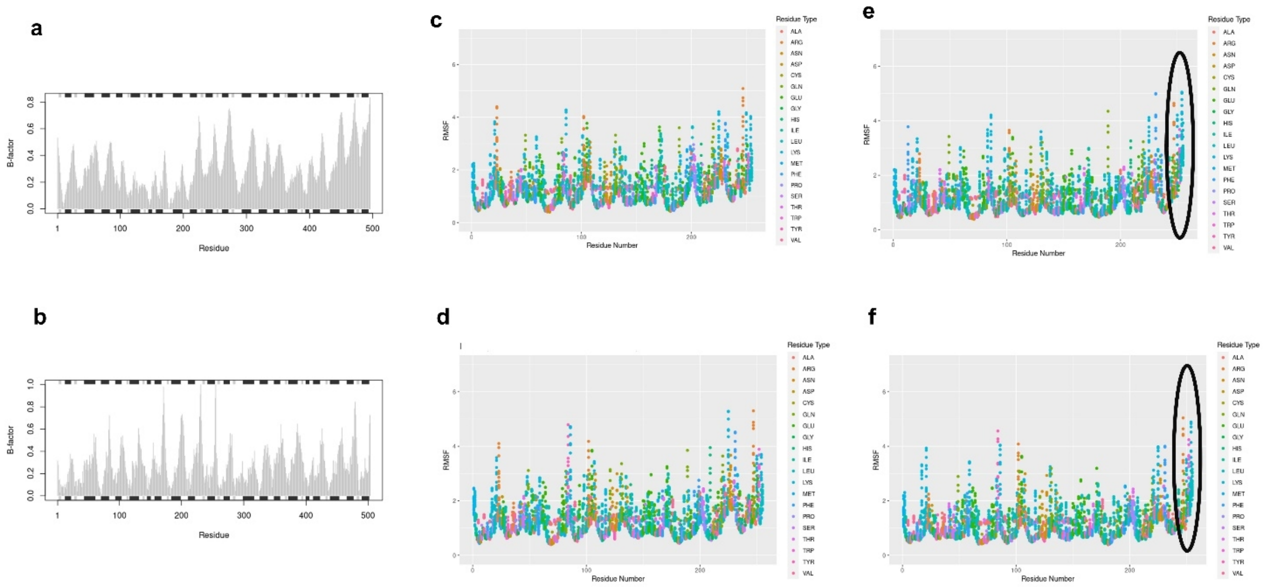

Supplementary Figure 6.

All panels are plotted as function of residue number. **A)** Normalized B-factors for inhibitor-free system (**2JFX**: GR-D-Glu). **B)** Normalized B-factors for inhibitor-bound structure (**2JFZ**: GR-D-Glu-Compound-A). **C)** RMSF (Å) for monomer A of inhibitor-bound system (**GR-D-Glu-Compound A**). **D)** RMSF (Å) for monomer B of inhibitor-bound system (**GR-D-Glu-Compound A**). **E)** RMSF (Å) for monomer A of inhibitor-free system (**GR-D-Glu**). **F)** RMSF (Å) for monomer B of inhibitor-free system (**GR-D-Glu**). The oval highlights residues the uninhibited state of GR shows increased flexibility, specifically in residues 240-255 which form the C-terminal α-helix and a major part of the allosteric inhibitor binding site.

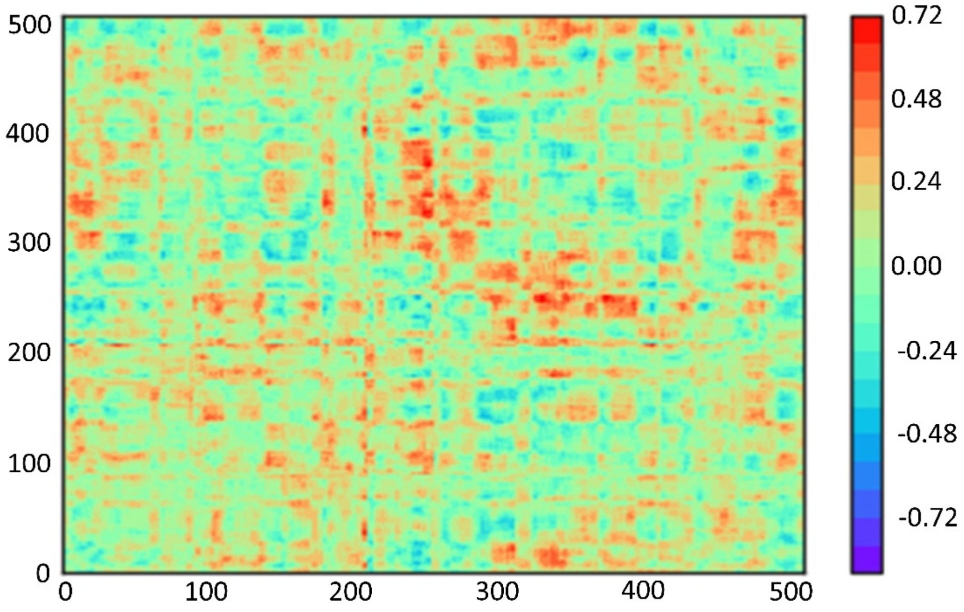

Supplementary Figure 7.

Topology map of the difference between the DCCM for the uninhibited system (**GR-D-Glu**) and the inhibited system (**GR-D-Glu-Compound A**). The difference DCCM topology map, from subtracting the DCCM for **GR-D-Glu-compound A** from the DCCM for the native **GR-D-Glu**. Positive changes in coupled motion represent motions that are lost upon binding of the allosteric inhibitor, compound A. The salient pattern is very similar to what is seen for NP-020560, showing a loss in coupled motion between the monomers.

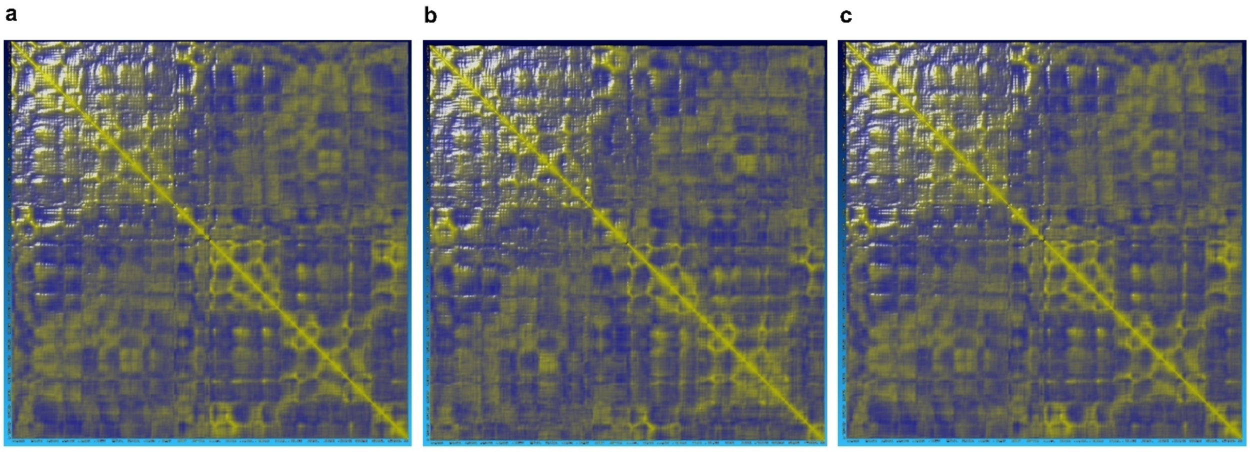

Supplementary Figure 8.

A) DCCM map for uninhibited system (**GR-D-Glu**). **B)** DCCM map for inhibited system (**GR-D-Glu-NP 020560**). **C)** DCCM map for inhibited system (**GR-D-Glu-Compound A**).

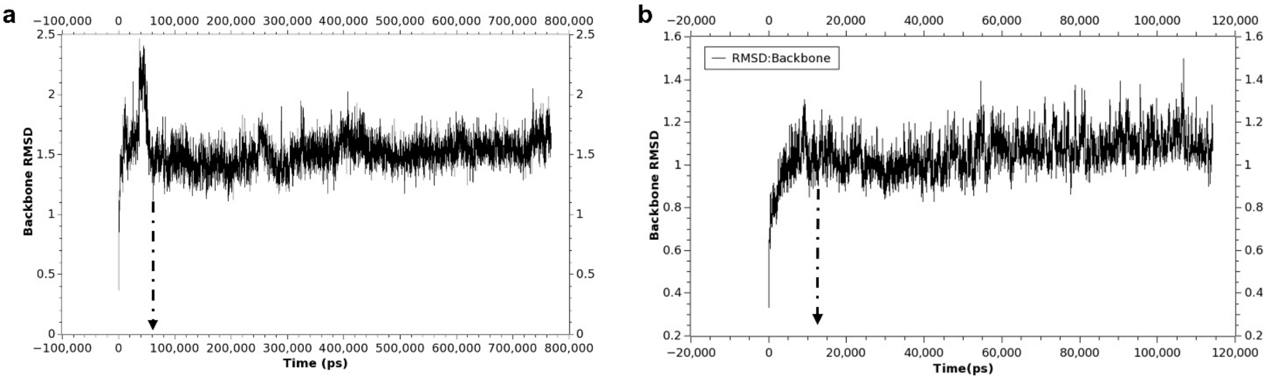

Supplementary Figure 9.

Root Mean Square Deviation (RMSD) of amino acid backbone during Molecular Dynamic simulation run for **A.** 2JFZ and **B**. 4B1F. 2JFZ achieves equilibrium around 60 ns while 4B1F achieves equilibrium around 10 ns.

Supplementary Table

Smiles Strings	Active or Decoy
CC(C)CN1C(=O)N(C)C(=O)c2c1nn(Cc3cccc4ccccc34)c2c5ccncc5	Active
CN1C(=O)c2c(nn(Cc3ccnc4ccc(Cl)cc34)c2c5cc(C#N)cn5C)N(CC6CC6)C1=O	Active
CN1C(=O)c2c(nn(Cc3ccnc4ccc(Cl)cc34)c2c5ccc(o5)S(C)(=O)=O)N(CC6CC6)C1=O	Active
CN1C(=O)c2c(nn(Cc3ccnc4ccc(Cl)cc34)c2c5oc(cc5Cl)S(C)(=O)=O)N(CC6CC6)C1=O	Active
Cc1oc(cc1c2c3C(=O)N(C)C(=O)N(CC4CC4)c3nn2Cc5ccnc6ccc(Cl)cc56)S(C)(=O)=O	Active
Cc1oc(cc1c2c3C(=O)N(C)C(=O)N(CC4CC4)c3nn2Cc5cn(C)c6ccc(Cl)cc56)S(C)(=O)=O	Active
CN1C(=O)c2c(nn(Cc3c[nH]c4ccc(Cl)cc34)c2c5nc(cn5C)S(N)(=O)=O)N(CC6CC6)C1=O	Active
CN1C(=O)c2c(nn(Cc3ccnc4ccc(Cl)cc34)c2c5cc(cn5C)S(N)(=O)=O)N(CC6CC6)C1=O	Active
CNS(=O)(=O)c1cc(c2c3C(=O)N(C)C(=O)N(CC4CC4)c3nn2Cc5ccnc6ccc(Cl)cc56)n(C)c1	Active
CNS(=O)(=O)c1cc(c2c3C(=O)N(C)C(=O)N(CC4CC4)c3nn2Cc5c[nH]c6ccc(Cl)cc56)n(C)c1	Active
CONS(=O)(=O)c1cc(c2c3C(=O)N(C)C(=O)N(CC4CC4)c3nn2Cc5c[nH]c6ccc(Cl)cc56)n(C)c1	Active
CONS(=O)(=O)c1cc(c2c3C(=O)N(C)C(=O)N(CC4CC4)c3nn2Cc5ccnc6ccc(Cl)cc56)n(C)c1	Active
CN1C(=O)c2c(nn(Cc3ccnc4ccc(Cl)cc34)c2c5nc(cn5C)S(C)(=O)=O)N(CC6CC6)C1=O	Active
CN1C(=O)c2c(nn(Cc3c[nH]c4ccc(Cl)cc34)c2c5nc(cn5C)S(C)	Active

(=O)=O)N(CC6CC6)C1=O	
CN1C(=O)c2c(nn(Cc3ccnc4ccc(Cl)cc34)c2c5cc(cn5C)S(C)=O)N(CC6CC6)C1=O	Active
CN1C(=O)c2c(nn(Cc3c[nH]c4ccc(Cl)cc34)c2c5cc(cn5C)S(C)=O)N(CC6CC6)C1=O	Active
CN1C(=O)c2c(nn(Cc3ccnc4ccc(Cl)cc34)c2c5cc(cn5C)S(C)(=O)=O)N(CC6CC6)C1=O	Active
CN1C(=O)c2c(nn(Cc3c[nH]c4ccc(Cl)cc34)c2c5cc(cn5C)S(C)<br>(=O)=O)N(CC6CC6)C1=O	Active
CN1C(=O)c2c(nn(Cc3cn(C)c4ccc(Cl)cc34)c2c5cc(cn5C)S(C)<br>(=O)=O)N(CC6CC6)C1=O	Active
CC(=O)c1cc(c2c3C(=O)N(C)C(=O)N(CC4CC4)c3nn2Cc5ccnc6ccc(Cl)cc56)n(C)c1	Active
CN1C(=O)c2c(nn(Cc3ccnc4ccc(Cl)cc34)c2c5cncn5C)N(CC6CC6)C1=O	Active
CN1C(=O)c2c(nn(Cc3ccnc4ccc(Cl)cc34)c2c5nc(C#N)cn5C)N(CC6CC6)C1=O	Active
Cc1cc(C#N)oc1c2c3C(=O)N(C)C(=O)N(CC4CC4)c3nn2Cc5ccnc6ccc(Cl)cc56	Active
CC(C)CN1C(=O)N(C)C(=O)c2c1nn(Cc3ccnc4ccc(Cl)cc34)c2c5cc(C#N)cn5C	Active
CC(C)CN1C(=O)N(C)C(=O)c2c1nn(Cc3ccnc4ccc(Cl)cc34)c2c5cncn5C	Active
Cc1oc(C#N)cc1c2c3C(=O)N(C)C(=O)N(CC4CC4)c3nn2Cc5ccnc6ccc(Cl)cc56	Active
Cc1c[nH]nc1c2c3C(=O)N(C)C(=O)N(CC4CC4)c3nn2Cc5ccnc6ccc(Cl)cc56	Active
CN1C(=O)c2c(nn(Cc3ccnc4ccc(Cl)cc34)c2c5nncn5C)N(CC6CC6)C1=O	Active
CC(C)CN1C(=O)N(C)C(=O)c2c1nn(Cc3ccnc4ccc(Cl)cc34)c2c5cccn5C	Active
Cc1sc(N)nc1c2c3C(=O)N(C)C(=O)N(CC4CC4)c3nn2Cc5ccnc6ccc(Cl)cc56	Active
Cc1nc(N)sc1c2c3C(=O)N(C)C(=O)N(CC4CC4)c3nn2Cc5ccnc6ccc(Cl)cc56	Active
CN1C(=O)c2c(nn(Cc3ccnc4ccc(Cl)cc34)c2c5ncnn5C)N(CC6CC6)C1=O	Active
Cc1nc[nH]c1c2c3C(=O)N(C)C(=O)N(CC4CC4)c3nn2Cc5ccnc6ccc(Cl)cc56	Active
C#CCN1C(=O)c2c(nn(Cc3ccnc4ccc(Cl)cc34)c2c5cncn5C)N(CC6CC6)C1=O	Active
Cn1cncc1c2c3C(=O)N(CC#CCO)C(=O)N(CC4CC4)c3nn2Cc5ccnc6ccc(Cl)cc56	Active
C=CCN1C(=O)c2c(nn(Cc3ccnc4ccc(Cl)cc34)c2c5cncn5C)N(CC6CC6)C1=O	Active
CC#CCN1C(=O)c2c(nn(Cc3ccnc4ccc(Cl)cc34)c2c5cncn5C)N(CC6CC6)C1=O	Active
Cn1cncc1c2c3C(=O)N(CC4CC4)C(=O)N(CC5CC5)c3nn2Cc6ccnc7ccc(Cl)cc67	Active
CCN1C(=O)c2c(nn(Cc3ccnc4ccc(Cl)cc34)c2c5cncn5C)N(CC6CC6)C1=O	Active
Cn1cncc1c2c3C(=O)N(C(=O)N(CC4CC4)c3nn2Cc5ccnc6ccc(Cl)cc56)C7CC7	Active
Cn1cncc1c2c3C(=O)N(CC#CCN4CCOCC4)C(=O)N(CC5CC5)c3nn2Cc6ccnc7ccc(Cl)<br>cc67	Active
CC(C)N1C(=O)c2c(nn(Cc3ccnc4ccc(Cl)cc34)c2c5cncn5C)N(CC6CC6)C1=O	Active
C#CC(C)N1C(=O)c2c(nn(Cc3ccnc4ccc(Cl)cc34)c2c5cncn5C)N(CC6CC6)C1=O	Active
Cn1cc(C#N)cc1c2c3C(=O)N(CC#CCn4cncn4)C(=O)N(CC5CC5)c3nn2Cc6ccnc7ccc(C<br>l)cc67	Active
C#CCN1C(=O)c2c(nn(Cc3ccnc4ccc(Cl)cc34)c2c5cc(C#N)cn5C)N(CC6CC6)C1=O	Active
Cn1cc(C#N)cc1c2c3C(=O)N(CC#CCN4CCOCC4)C(=O)N(CC5CC5)c3nn2Cc6ccnc7c<br>cc(Cl)cc67	Active
Cn1cc(C#N)cc1c2c3C(=O)N(CC#N)C(=O)N(CC4CC4)c3nn2Cc5ccnc6ccc(Cl)cc56	Active
Cn1cc(C#N)cc1c2c3C(=O)N(CC#CCO)C(=O)N(CC4CC4)c3nn2Cc5ccnc6ccc(Cl)cc56	Active
Cn1cc(C#N)cc1c2c3C(=O)N(CC4CC4)C(=O)N(CC5CC5)c3nn2Cc6ccnc7ccc(Cl)cc67	Active
CCN1C(=O)c2c(nn(Cc3ccnc4ccc(Cl)cc34)c2c5cc(C#N)cn5C)N(CC6CC6)C1=O	Active
C=CCN1C(=O)c2c(nn(Cc3ccnc4ccc(Cl)cc34)c2c5cc(C#N)cn5C)N(CC6CC6)C1=O	Active
CC#CCN1C(=O)c2c(nn(Cc3ccnc4ccc(Cl)cc34)c2c5cc(C#N)cn5C)N(CC6CC6)C1=O	Active
Cn1cc(C#N)cc1c2c3C(=O)N(C(=O)N(CC4CC4)c3nn2Cc5ccnc6ccc(Cl)cc56)C7CC7	Active
Cn1cc(C#N)cc1c2c3C(=O)N(CCO)C(=O)N(CC4CC4)c3nn2Cc5ccnc6ccc(Cl)cc56	Active
Cn1cc(C#N)cc1c2c3C(=O)N(CC#CCn4cccn4)C(=O)N(CC5CC5)c3nn2Cc6ccnc7ccc(C<br>l)cc67	Active
C#CC(C)N1C(=O)c2c(nn(Cc3ccnc4ccc(Cl)cc34)c2c5cc(C#N)cn5C)N(CC6CC6)C1=O	Active
CC(C)N1C(=O)c2c(nn(Cc3ccnc4ccc(Cl)cc34)c2c5cc(C#N)cn5C)N(CC6CC6)C1=O	Active
Cn1cc(C#N)cc1c2c3C(=O)N(CC#CCn4ccnc4)C(=O)N(CC5CC5)c3nn2Cc6ccnc7ccc(C<br>l)cc67	Active

Cn1cc(C#N)cc1c2c3C(=O)N(CCn4cccn4)C(=O)N(CC5CC5)c3nn2Cc6ccnc7ccc(Cl)cc67	Active
Cn1cc(C#N)cc1c2c3C(=O)N(CCn4ccnn4)C(=O)N(CC5CC5)c3nn2Cc6ccnc7ccc(Cl)cc67	Active
C#CCN1C(=O)c2c(nn(Cc3ccnc4ccc(Cl)cc34)c2c5nc(C#N)cn5C)N(CC6CC6)C1=O	Active
Cn1cc(C#N)nc1c2c3C(=O)N(CC4CC4)C(=O)N(CC5CC5)c3nn2Cc6ccnc7ccc(Cl)cc67	Active
CCN1C(=O)c2c(nn(Cc3ccnc4ccc(Cl)cc34)c2c5nc(C#N)cn5C)N(CC6CC6)C1=O	Active
C=CCN1C(=O)c2c(nn(Cc3ccnc4ccc(Cl)cc34)c2c5nc(C#N)cn5C)N(CC6CC6)C1=O	Active
Cn1cc(C#N)nc1c2c3C(=O)N(C(=O)N(CC4CC4)c3nn2Cc5ccnc6ccc(Cl)cc56)C7CC7	Active
CC1CC(C)CN(C1)C(=O)COC(=O)c2ccc(cc2)N3C(=O)c4ccccc4C3=O	Decoy
CN(C(=O)COC(=O)C=Cc1ccc(cc1)S(=O)(=O)N2CCc3ccccc23)C4CCS(=O)(=O)C4	Decoy
CCOc1cc(ccc1O)C2C3=C(Oc4ccc(Br)cc4C3=O)C(=O)N2Cc5ccc6OCOc6c5	Decoy
CCS(=O)(=O)c1cccc(c1)C(=O)N(Cc2ccco2)c3nc4ccc(cc4s3)S(C)(=O)=O	Decoy
Cc1ccc(cc1)S(=O)(=O)N2CCN(CC2)C(=O)COC(=O)c3ccc(cc3)C(=O)c4ccccc4	Decoy
O=C(NNS(=O)(=O)c1cccc2nsnc12)c3ccccc3NS(=O)(=O)c4cccs4	Decoy
N=C1C(=Cc2ccc(cc2)OCc3ccc4OCOc4c3)C(=O)N=C5SC(=CC(=O)N6CCCC6)NN15	Decoy
CCOC(=O)N1CCN2c3ccc(cc3CC4(C(=O)NC(=O)N(CCc5ccccc5)C4=O)C2C1)[N+](=O)[O-]	Decoy
COC1=CN(CC(=O)Nc2cccc(c2)C(F)(F)F)C(=CC1=O)CN3CCN(CC3)C(=O)c4ccco4	Decoy
COC(=O)C1=C2SC(=Cc3ccc(OC)c(c3)OC)C(=O)N2C(N)=C(C(=O)OC)C1c4ccc(OC)c(c4)OC	Decoy
CC(=O)OCC1OC(OC2=CC(=O)c3c(O)cccc3C2=O)C(OC(C)=O)C(OC(C)=O)C1OC(C)=O	Decoy
COc1ccc(cc1)N2CC(CC2=O)c3nnc(NC(=O)C4CC(=O)N(C4)c5cccc(C)c5C)s3	Decoy
COc1ccc(c(c1)OC)N2CC(CC2=O)C(=O)N3CCN(CC3)C(=O)C4COc5ccccc5O4	Decoy
CCN(CC)S(=O)(=O)c1ccc(cc1)C(=O)N2CCN(Cc3nc(cs3)c4ccc(F)cc4)CC2	Decoy
COc1cc(C=C2C(=N)N3N=CSC3=NC2=O)cc(Br)c1OC(=O)c4cccs4	Decoy
COc1ccc(c(c1)OC)N2CC(CC2=O)C(=O)N3CCN(CC3)S(=O)(=O)c4ccc(F)c(F)c4	Decoy
COc1ccccc1C(=O)NC(C(=O)N2CCN(CC2)C(=O)c3ccco3)S(=O)(=O)c4ccccc4	Decoy
COc1ccc(cc1)CN(CC2=Cc3ccc(C)c(C)c3NC2=O)S(=O)(=O)c4ccc5OCCOc5c4	Decoy
Cc1ccccc1c2ccc(nn2)N3CCN(CC3)C(=O)c4ccc(cc4)S(=O)(=O)N5CCCC5	Decoy
O=C(COc1ccccc1F)N2CCN(CC3=CC(=O)N4N=C(SC4=N3)c5ccccc5F)CC2	Decoy
O=C(C1Cn2ccnc2C3(CCN(CC3)S(=O)(=O)c4ccccc4)O1)N5CCCCC5	Decoy
O=C(c1noc2ccc(cc12)S(=O)(=O)N3CC[NH+](Cc4ccccc4)CC3)N5CCCC5	Decoy
CN(C(=O)COC(=O)C1CCN(CC1)S(=O)(=O)c2ccc3OCCOc3c2)c4ccccc4	Decoy
COc1ccc2OC(c3ccc(OC)c(c3)OC)C4C(=Nc5ncnn5C4c6ccc(F)cc6)c2c1	Decoy
COc1cccc(c1)N2CC(CC2=O)C(=O)Oc3cccc(c3)N4C(=O)C5CC=CC(C)C5C4=O	Decoy
O=C(N=C1SC=CN1Cc2ccccc2F)C3=NN(C(=O)CC3)C4CCS(=O)(=O)C4	Decoy
C[NH+](C)CCCN(C(=O)C1CCN(CC1)c2nc3ccccc3o2)C4CCS(=O)(=O)C4	Decoy
COc1cc(cc(OC)c1OC)C2C(C(=O)N2c3ccc(C)cc3)N4C(=O)c5ccccc5C4=O	Decoy
COc1ccc(OC)c(c1)C2CC(=O)C3=C(C2)NC(=O)CC3c4cc(ccc4Cl)[N+](=O)[O-]	Decoy
COc1ccc(NC(=O)CN2C=Nc3c(oc4nc(c5CCCc5c34)C(C)C)C2=O)c(c1)OC	Decoy
COc1cccc(c1)N2CCN(CC2)C(=O)C3CC(=O)N(C3)c4cccc(c4)c5noc(C)n5	Decoy
O=C(c1nc(c2cccs2)n(n1)c3cccc(F)c3)N4CCCC4c5nnc6ccccn56	Decoy
CC(=O)Nc1ccc(cc1)NC(=O)CN2C(=O)C(Sc3ccccc23)C(=O)N4CCCCCC4	Decoy
COC(=O)[C@@]1(N[C@@H](c2ccc(O)c(c2)OC)[C@H]3C(=O)N(C(=O)[C@H]13)c4ccccc4F)c5ccccc5	Decoy
CC(C)(C)OC(=O)N1CCC(CC1)C(=O)N2CCN(CC2)C(=O)C3COc4ccccc4O3	Decoy
Cc1cccc(NC(=O)CN2N=NC3C(=O)N(C(=O)C23)c4ccc(Br)cc4)c1C	Decoy
CS(=O)(=O)N1CCN(CC1)C(=O)C2CCN(CC2)S(=O)(=O)c3ccc4CCCCc4c3	Decoy
COc1ccc(cc1)N(Cc2cccs2)C(=O)C[NH+]3CCN(CC(=O)N4CCOCC4)CC3	Decoy
O=C(CSc1nnc(s1)N2CCN(CC2)C(=O)Cc3noc4ccccc34)NCc5ccco5	Decoy

O=C(NC1CCN(CC1)c2ccccc2C(=O)N3CC[NH+](CC3)C4CCCC4)c5ccc6OCOc6c5	Decoy
COc1ccc(cc1)S(=O)(=O)N2CCOC23CCN(CC3)S(=O)(=O)c4ccccc4Cl	Decoy
CCOc1cccc2sc(nc12)N(CCC[NH+]3CCOCC3)C(=O)C4CC(=O)N(C4)c5ccc(F)cc5	Decoy
C[C@@H]1CN(C[C@@H](C)O1)C2=C(/C=C3\ SC(=S)N(Cc4ccco4)C3=O)C(=O)N5C=CC=CC5=N2	Decoy
Cc1ccccc1C2c3ccsc3CC[NH+]2CC(=O)N4CCN(C(=O)c5ccc(cc5)[N+](=O)[O-])C(C)C4	Decoy
COc1ccc(cc1)N2CCN(CC2)C(=O)CSc3nnc(N4CCCC4)n3c5ccc(C)cc5	Decoy
CCCCCN1CC(=O)N2C(CC3=C4C=CC=CC4=[NH+]C3C2c5cc(OC)c(OC)c(c5)OC)C1=O	Decoy
COc1cccc2C=C(C(=O)N3CCN(CC3)C4=NN=C(CS4)c5ccccc5)C(=O)Oc12	Decoy
CCOCCCN1CC(=O)N2C(CC3=C4C=CC=CC4=[NH+]C3C2c5ccc(cc5)[N+](=O)[O-])C1=O	Decoy
COc1ccc(cc1)N2C(=O)CCC(C(=O)N3CCN(CC3)C(=O)C4CCCO4)C2c5ccccc5	Decoy
COc1ccc2nc(sc2c1)N(CCC[NH+]3CCOCC3)C(=O)C4COc5ccccc5O4	Decoy
COC(=O)C1=C2SC(=Cc3cccc(c3)OC)C(=O)N2C(=N)C(C1c4cccc(c4)OC)S(=O)(=O)c5ccccc5	Decoy
CCC[NH+]1CCc2c(C1)sc(NC(=O)c3ccc(cc3)S(=O)(=O)N4CC(C)CC(C)C4)c2C(=O)OC	Decoy
COc1ccc(OC)c2sc(nc12)N(Cc3cccnc3)C(=O)C4Oc5ccccc5OC4C	Decoy
COc1cccc(c1OC)C2C3[NH+]=C4C=CC=CC4=C3CC5C(=O)N(CCCOC(C)C)CC(=O)N25	Decoy
CC1Cc2ccccc2N1S(=O)(=O)c3cccc(c3)C(=O)N4CCN(CC4)C(=O)c5ccco5	Decoy
C=CC[NH+](CC(=O)N1CCN(CC1)S(=O)(=O)c2ccc3OCCCOc3c2)Cc4cccs4	Decoy
CCOc1ccc(cc1)N(CC(=O)Nc2cccc(c2)N(C)S(C)(=O)=O)S(=O)(=O)c3ccc(C)cc3	Decoy
CCCN1C(=O)c2ccccc2[C@@H](C(=O)N3CCN(CC3)C4CCCC4)[C@H]1c5cc(OC)c(OC)c(c5)OC	Decoy
CCOc1ccccc1N2C(=O)C3C4C=C(C(=O)Oc5ccccc5)C(C3C2=O)C6C(=O)N(C(=O)C46)c7ccccc7OCC	Decoy
COc1cc2CCN3C(=O)c4ccccc4[C@H](C(=O)N5CCN(Cc6ccc7OCOc7c6)CC5)[C@H]3c2cc1OC	Decoy
CS(=O)(=O)C1=NN2C(=NC(=O)C(=Cc3ccc(OC(=O)c4cccs4)c(Br)c3)C2=N)S1	Decoy
Cc1cnc(cn1)C(=O)N(CCC(=O)N2CCN(CC2)C(c3ccccc3)c4ccccc4)Cc5ccco5	Decoy
O=C(CSc1nnc(c2ccccc2F)n1C3CCCCC3)N4CCN(CC4)C(=O)c5ccco5	Decoy
CC(C)c1ccc(cc1)N(CC(=O)N2CCN(CC2)C(=O)C3CCCO3)S(=O)(=O)c4ccccc4	Decoy
CCOC(=O)C1=C(C)C2C3C(=O)N(C(=O)C3C1(C)C4C(=O)N(C(=O)C24)c5ccccc5F)c6ccccc6F	Decoy
CCOc1ccccc1N2CC(CC2=O)C(=O)Nc3cc(ccc3OC)S(=O)(=O)N4CCCCC4	Decoy
COc1cc(ccc1OC(C)C)C2C(=O)N(CC(=O)N2CC3COc4ccccc4O3)C5CCCCC5	Decoy
CCOc1ccc(cc1)NC(=O)CN2C(=O)C(=C3SC(=S)N(C3=O)C4CCS(=O)(=O)C4)c5ccccc25	Decoy
COc1cc(C=C2C(=N)N3N=CSC3=NC2=O)cc(Br)c1OC(=O)c4cccs4	Decoy
COc1ccc(cc1)N2CCN(CC2)C(=O)C3CCN(CC3)S(=O)(=O)c4cccc5cccnc45	Decoy
COC1OC2CCC(OC2C(OS(=O)(=O)c3ccc(C)cc3)C1OS(=O)(=O)c4ccc(C)cc4)c5ccccc5	Decoy
CC[NH+](CC)CCCN1C(=O)C2=C(C(=O)c3cc(Br)ccc3O2)C1c4cc(OC)c(OC)c(c4)OC	Decoy
CCOc1ccc(cc1OCC)N2CC(CNC(=O)c3ccc(cc3)S(=O)(=O)N4CCCCC4)OC2=O	Decoy
CC(=O)Nc1ccc(cc1)S(=O)(=O)N2CCN(CC2)c3c(cc(cc3[N+](=O)[O-])C(F)(F)F)[N+](=O)[O-]	Decoy
COc1cc(C(=O)N2CCC3(CC2)OCCN3S(=O)(=O)c4ccc(C)cc4)c(cc1OC)[N+](=O)[O-]	Decoy
CCOC(=O)C1CCCN(C1)C(=O)C2CCN(CC2)S(=O)(=O)c3c(C)noc3C=Cc4ccc(C)cc4	Decoy
COC(=O)CN1C(=O)SC2=C1N(C(=O)CC2c3ccc(OC)c(c3)OC)c4cccc(c4)OC	Decoy
CCOC(=O)c1ccc2c(c1)SC(=NC(=O)c3ccc(cc3)S(=O)(=O)N(C)C)N2CC(=O)OC	Decoy
COc1ccc(cc1OC)C2CC(=NN2C(=O)CSc3nnc(o3)c4ccco4)c5cccs5	Decoy

Supplementary Table 1. SMILES strings for the library of 65 active pyrazolopyridiminedione analogs and respective inactive/decoy compounds generated using the Database of Useful Decoys – Enhanced (DUD–E) website that were used for ROC analysis.

References:

1. Rej, R. A convenient continuous-rate spectrophotometric method for determination of amino acid substrate specificity of aminotransferases: application to isoenzymes of aspartate aminotransferase. *Analytical biochemistry* **119**, 205-210 (1982).
